# Expanding structural diversity of 5′-aminouridine moiety of sansanmycin via mutational biosynthesis

**DOI:** 10.3389/fbioe.2023.1278601

**Published:** 2023-10-30

**Authors:** Yuan Lu, Yihong Li, Jiahui Fan, Xingxing Li, Hongmin Sun, Lifei Wang, Xingli Han, Yuting Zhu, Tianyu Zhang, Yuanyuan Shi, Yunying Xie, Bin Hong

**Affiliations:** ^1^ CAMS Key Laboratory of Synthetic Biology for Drug Innovation and NHC Key Laboratory of Biotechnology of Antibiotics, Institute of Medicinal Biotechnology, Chinese Academy of Medical Sciences and Peking Union Medical College, Beijing, China; ^2^ State Key Laboratory of Respiratory Disease, Guangzhou Institutes of Biomedicine and Health (GIBH), Chinese Academy of Sciences (CAS), Guangzhou, China; ^3^ China-New Zealand Joint Laboratory of Biomedicine and Health, Guangzhou Institutes of Biomedicine and Health (GIBH), Chinese Academy of Sciences (CAS), Guangzhou, China; ^4^ Guangdong-Hong Kong-Macau Joint Laboratory of Respiratory Infectious Diseases, Guangzhou, China; ^5^ University of Chinese Academy of Sciences (UCAS), Beijing, China

**Keywords:** sansanmycin, 5′-aminouridine, structural diversity, mutational biosynthesis, new analogues

## Abstract

Sansanmycins represent a family of uridyl peptide antibiotics with antimicrobial activity specifically against *Mycobacterium tuberculosis* (including drug-resistant *M. tuberculosis*) and *Pseudomonas aeruginosa*. They target translocase I (MraY) to inhibit bacterial cell wall assembly. Given the unique mechanism of action, sansanmycin has emerged as a potential lead compound for developing new anti-tuberculosis drugs, while the 5′-aminouridine moiety plays a crucial role in the pharmacophore of sansanmycin. For expanding the structural diversity of the 5′-aminouridine moiety of sansanmycin through biosynthetic methods, we firstly demonstrated that SsaM and SsaK are responsible for the biosynthesis of the 5′-aminouridine moiety of sansanmycin *in vivo*. Using the *ssaK* deletion mutant (SS/KKO), we efficiently obtained a series of new analogues with modified 5′-aminouridine moieties through mutational biosynthesis. Based on molecular networking analysis of MS/MS, twenty-two new analogues (SS-KK-1 to -13 and SS-KK-A to -I) were identified. Among them, four new analogues (SS-KK-1 to -3 and SS-KK-C) were purified and bioassayed. SS-KK-2 showed better antibacterial activity against *E*. *coli ΔtolC* than the parent compound sansanmycin A. SS-KK-3 showed the same anti-TB activity as sansanmycin A against *M. tuberculosis* H37Rv as well as clinically isolated, drug-sensitive and multidrug-resistant *M. tuberculosis* strains. Furthermore, SS-KK-3 exhibited significantly improved structural stability compared to sansanmycin A. The results suggested that mutasynthesis is an effective and practical strategy for expanding the structural diversity of 5′-aminouridine moiety in sansanmycin.

## 1 Introduction

Tuberculosis (TB), an infectious disease caused by *Mycobacterium tuberculosis*, remains one of the most significant global health threats. The recently published “2022 Global Tuberculosis Report” (World Health Organization, 2022) points that TB is the second deadliest infectious disease following COVID-19. The COVID-19 pandemic has reversed the progress made against TB in recent years. Global TB deaths increased from 1.4 million in 2019 to 1.5 million in 2020 and further to 1.6 million in 2021. In the near future, TB may surpass COVID-19 again as the leading cause of death worldwide from a single infectious source as COVID-19 is gradually under control. The current approach to treating TB involves a 6-month course of quadruple therapy consisting of rifampicin (RIF), isoniazid (INH), ethambutol, and pyrazinamide (Zumla et al., 2013). While this treatment can effectively control drug-sensitive TB, it is ineffective against drug-resistant TB infections, which are becoming increasingly prevalent worldwide. Given the severity of the current TB situation and the propensity for drug resistance, the development of new drugs specifically targeting drug-resistant TB is urgently needed.

Sansanmycins (Xie et al., 2007), produced by *Streptomyces* sp. SS, belong to the family of uridyl peptide antibiotics (UPAs) including pacidamycins (Karwowski et al., 1989), napsamycins (Chatterjee et al., 1994), and mureidomycins (Inukai et al., 1989). They share a core scaffold of a 3′-deoxyuridine unit linked to a pseudo-tetra/pentapeptidyl backbone via an unusual enamide linkage. UPAs possess noteworthy antimicrobial activity against *Mycobacterium tuberculosis* (including drug-resistant *Mycobacterium tuberculosis*) (Shi et al., 2016) and *Pseudomonas aeruginosa* by targeting translocase I (MraY) to block bacterial cell wall assembly (Winn et al., 2010). As this unique mode of action has not been clinically targeted, UPA has emerged as a lead compound in the quest for new anti-TB drugs.

Extensive research has been conducted on UPAs’ biosynthesis logic and structural modification. UPAs are synthesized by a series of highly dissociated non-ribosomal peptide synthetases (NRPSs) (Zhang et al., 2010). Their assembly begins with the central building block 2,3-diaminobutyric acid (DABA), then the elongation of the peptide framework includes the attachment of the *N*-terminal amino acid (AA_1_) to the *β*-amino group of DABA, and the *C*-terminal ureidodipeptide (AA_3_ and AA_4_) linked to the *α*-amino group of DABA (Zhang et al., 2010; Zhang et al., 2011). The unusual ureidodipeptide assembly is achieved under the catalysis of a unique condensation enzyme PacN, resulting in the formation of a ureido-bond between AA_3_ and AA_4_ (Zhang et al., 2010; Ióca et al., 2021). The synthesis of 4′,5′-enamide-3′-deoxyuridine of UPA has been elucidated *in vitro*, which starts from uridine, and a flavin-dependent dehydrogenase Pac11 (i.e., PacK) catalyzes the oxidation of uridine to uridine-5′-aldehyde (Ragab et al., 2011). The aminotransferase Pac5 (i.e., PacE) catalyzes the transamination of the 5′-aldehyde group, and the dehydration reaction mediated by Pac13 (i.e., PacM) finally generates 4′,5′-enamide-3′-deoxyuridine (Ragab et al., 2011; Michailidou et al., 2017) (Supplementary Figure S1). Then under the catalysis of PacI, it is attached to pseudo-tetra backbone through an enamide bond, consequently producing UPAs (Zhang et al., 2011).

Previous structural modifications of UPAs mainly focused on substituting amino acid residues in peptide chains, such as obtaining *C*-terminally modified pacidamycin derivatives (Gruschow et al., 2009) and sansanmycin derivatives (Zhang et al., 2016) by precursor-directed biosynthesis. A series of new sansanmycin analogues with modification at *N*-terminal amino acid were obtained using mutational biosynthesis (Shi et al., 2016). Nevertheless, few analogue compounds showed significantly increase of the antibacterial activity. The 5′-aminouridine moiety in UPAs is believed to be essential for competing active site binding with the natural substrate UDP-*N*-acetylmuramoyl (UDP-MurNAc) pentapeptide of MraY (Walsh and Zhang, 2011). Recently, the MraY-inhibitor complex structure data show a common feature of the uridine binding pocket of naturally occurring nucleoside inhibitors including UPAs, and the residues that form the uridine binding pocket are likely involved in binding the natural substrate of MraY (Mashalidis et al., 2019). However, as an important part of pharmacophore, there have been no reported structural modifications of 5′-aminouridine through biosynthetic methods.

**FIGURE 1:**
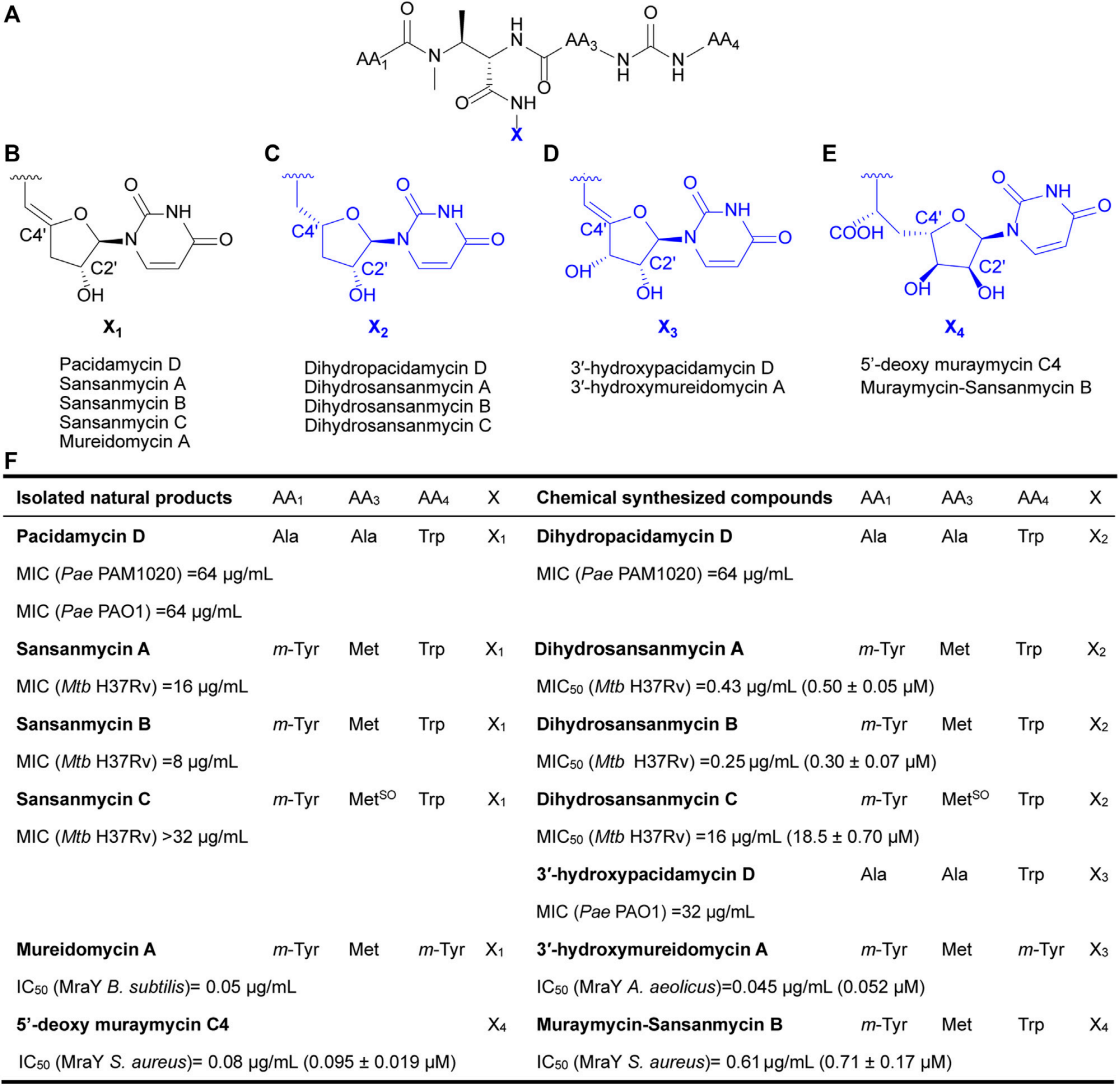
Chemical structures of natural UPAs (black) and synthetic analogues with modified 5′-aminouridine moiety (blue) and their corresponding inhibitory activity. **(A)** The pseudo-tetrapeptide backbone of UPAs. **(B)** The chemical structures of 5′-aminouridine of natural UPAs. **(C–E)** The chemical structures of modified 5′-aminouridine of synthetic analogues. **(F)** Detailed information on structure and inhibitory activities of natural UPAs and synthesized derivatives. *Pae*, *Pseudomonas aeruginosa*; *Mtb*, *Mycobacterium tuberculosis*; *B. subtilis*, *Bacillus subtilis*; *S. aureus*, *Staphylococcus aureus*; *A. aeolicus*, *Aquifex aeolicus*.

Currently, reported analogues with modified 5′-aminouridine are almost entirely obtained through chemical synthesis (Figure 1). Dihydropacidamycin D, the first analogue compound with modified 5′-aminouridine was obtained through the hydrogenation of C-4′ exocyclic olefin of pacidamycin D, and it showed comparable activity against *P. aeruginosa* to pacidamycin D (Boojamra et al., 2001) (Figures 1A–C,F), indicating that the hydrogenation of enamide linkage would not affect the activity of UPAs. In recent years, certain dihydrosansanmycin analogues with modified peptide chains have been reported to exhibit enhanced activity against *M. tuberculosis* (Tran et al., 2017; Tran et al., 2021) (Figures 1A–C, F). Notably, the stereochemistry at C-4′ of the ribose moiety in both dihydrosansanmycin and dihydropacidamycin has been suggested to be essential for antibacterial activity, with *S*-stereochemistry in analogues usually leading to loss or reduction of activity (Boojamra et al., 2001; Tran et al., 2017). The antibacterial activity of 3′-hydroxypacidamycin D against *P. aeruginosa* was comparable to that of pacidamycin D (Okamoto et al., 2012), while the MraY inhibition activity of 3′-hydroxymureidomycin A (Mashalidis et al., 2019) was also comparable to that of mureidomycin A (Inukai et al., 1993) (Figures 1A, D, F). These findings suggest that the introduction of a hydroxyl group at the 3′-position of the 5′-aminouridine moiety is well-tolerated for both MraY inhibition and antibacterial activity (Okamoto et al., 2012). In 2020, Niro et al. (Niro et al., 2020) reported a new hybrid structure, which consists of the peptide chain of sansanmycin B and uridine core moiety of 5′-deoxy muraymycin C4 (Figures 1A, E–F). Similar to UPA, the 5′-deoxy muraymycin C4 is also a uridine-derived nucleoside antibiotic, comprising a (6′*S*)-5′-deoxy-glycyluridine moiety, and demonstrates inhibitory activity against MraY (Koppermann et al., 2018). The muraymycin-sansanmycin B hybrid structure showed inhibition activity against MraY, but displayed no significant antibacterial activity against *P. aeruginosa* (Niro et al., 2020). This suggests that the hybrid structure resulting from the incorporation of uridine building blocks from other nucleoside antibiotics into UPAs may still exhibit inhibitory activity against MraY. Chemical synthesis of UPAs is challenging and low yielding, but it provides some insights into the structure-activity relationship of UPAs. What’s more, it indicates that making appropriate modifications to 5′-aminouridine can be further explored for new UPA derivatives to improve their activity and/or druggability.

Here, we aimed to expand the structural diversity of sansanmycin and explore the structure-activity relationship of 5′-aminouridine moiety by mutational biosynthesis. Within sansanmycin biosynthetic gene cluster, SsaM, SsaK and SsaE share high homology with PacM, PacK and PacE, respectively, which have been previously confirmed to be responsible for the biosynthesis of the 5′-aminouridine of pacidamycin *in vitro*. In this study, through *in vivo* assays, we demonstrated that SsaM and SsaK are responsible for the biosynthesis of 5′-aminouridine in sansanmycin biosynthetic pathway, and confirmed SsaM acts as the dehydratase by the detection of a new hydrated derivative. On this basis, two 5′-aminouridine analogues were fed to the knockout strains of *ssaK and ssaM*, and *ssaK* knockout strain showed higher yield of derivatives. Twenty-two new derivatives were identified by the molecular networking of LC-MS/MS data. Among them, four monomers were purified and one of them were further confirmed by NMR. SS-KK-2 exhibited improved antibacterial activity against *E. coli ΔtolC*. SS-KK-3 showed the same activity against *M. tuberculosis* including clinically isolated, multidrug-resistant strains with improved structural stability.

## 2 Results

### 2.1 Blocking the biosynthesis of 4′,5′-enamide-3′-deoxyuridine

**FIGURE 2:**
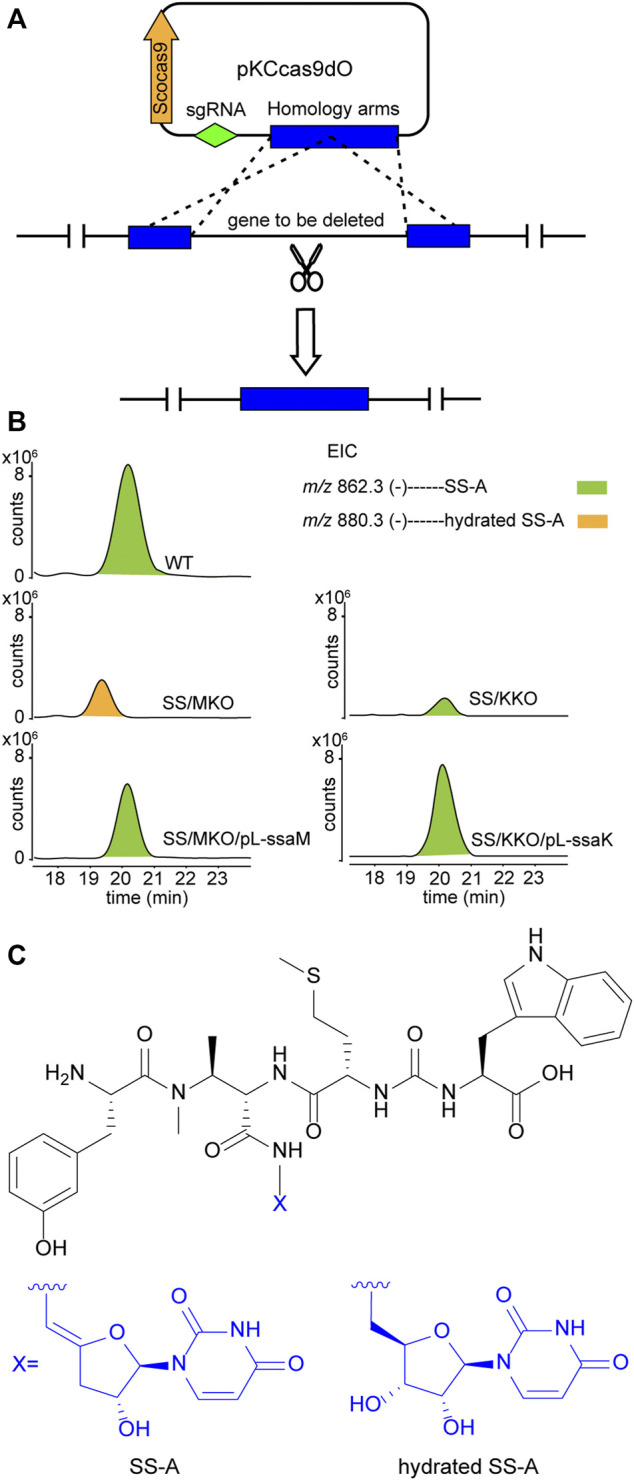
Effects of blocking 5′-aminouridine biosynthetic gene *ssaM* and *ssaK* on sansanmycin production. **(A)** Construction of mutant strains with deleted 5′-aminouridine biosynthetic genes. **(B)** LC-ESI-MS analyses of sansanmycin A (SS-A) and its derivatives in different strains. **(C)** Structures of SS-A and hydrated SS-A.

Upon scrutiny of the sansanmycin biosynthetic gene cluster, SsaM, SsaK, and SsaE were found to exhibit over 80% amino acid sequence identity with PacM, PacK, and PacE (Supplementary Figures S2–4), respectively, indicating their probable role in the biosynthesis of the 5′-aminouridine moiety in sansanmycin. To investigate the function of SsaM, SsaK, and SsaE *in vivo*, the corresponding encoding genes *ssaM*, *ssaK*, and *ssaE* were in-frame deleted from *Streptomyces* sp. SS by CRISPR/Cas9 using plasmid pKCcas9dO (He et al., 2015), which contains codon-optimized *scocas9* encoding *Streptococcus* pyogenes Cas9 protein. Deletion plasmids pKC-M, pKC-K and pKC-E were constructed by replacing the sgRNA sequence and homology arm sequences in plasmid pKCcas9dO with the corresponding sequences of the genes to be knocked out, and were further introduced into *Streptomyces* sp. SS by conjugation (Figure 2A). Knock-out mutants SS/MKO, SS/KKO and SS/EKO of *ssaM*, *ssaK*, and *ssaE* were verified by PCR, and the results showed that the correct SS/MKO and SS/KKO were obtained (Supplementary Figures S5, 6). There was no correct SS/EKO obtained even after many efforts, which may be due to the off-target effect. The complete coding regions of *ssaM* and *ssaK* were cloned into the expression plasmid pL646 (Hong et al., 2007) derived from pSET152 (Bierman et al., 19922) under the control of a strong constitutive promoter, *ermE*p*, respectively. Then resulting plasmids pL-ssaM and pL-ssaK were introduced into SS/MKO and SS/KKO by conjugation, respectively, and the complementary strains SS/MKO/pL-ssaM and SS/KKO/pL-ssaK were obtained to exclude the possibility of polar effects in SS/MKO and SS/KKO.

Secondary metabolites of SS/MKO and SS/KKO, as well as the complementary strains SS/MKO/pL-ssaM and SS/KKO/pL-ssaK were analyzed by LC-MS/MS, with wild-type strain as control. In SS/MKO, sansanmycin A (SS-A), the major component compound of wild-type strain, was eliminated and instead a new hydrated sansanmycin A (hydrated SS-A) was detected (Figures 2B, C, Supplementary Figure S7). The production of hydrated SS-A confirmed that SsaM acts as the dehydratase responsible for biosynthesizing the 5′-aminouridine of sansanmycins *in vivo*. Mutant SS/KKO still produced trace amount of SS-A (Figure 2B), potentially due to its function being partially complemented by other enzymes in the production strain. Production of sansanmycins could be restored in both complementary strains (Figure 2B). All of above results demonstrated that SsaM and SsaK were responsible for the biosynthesis of 5′-aminouridine in sansanmycin biosynthetic pathway *in vivo*.

### 2.2 Production of new sansanmycin analogues by mutational biosynthesis

**FIGURE 3:**
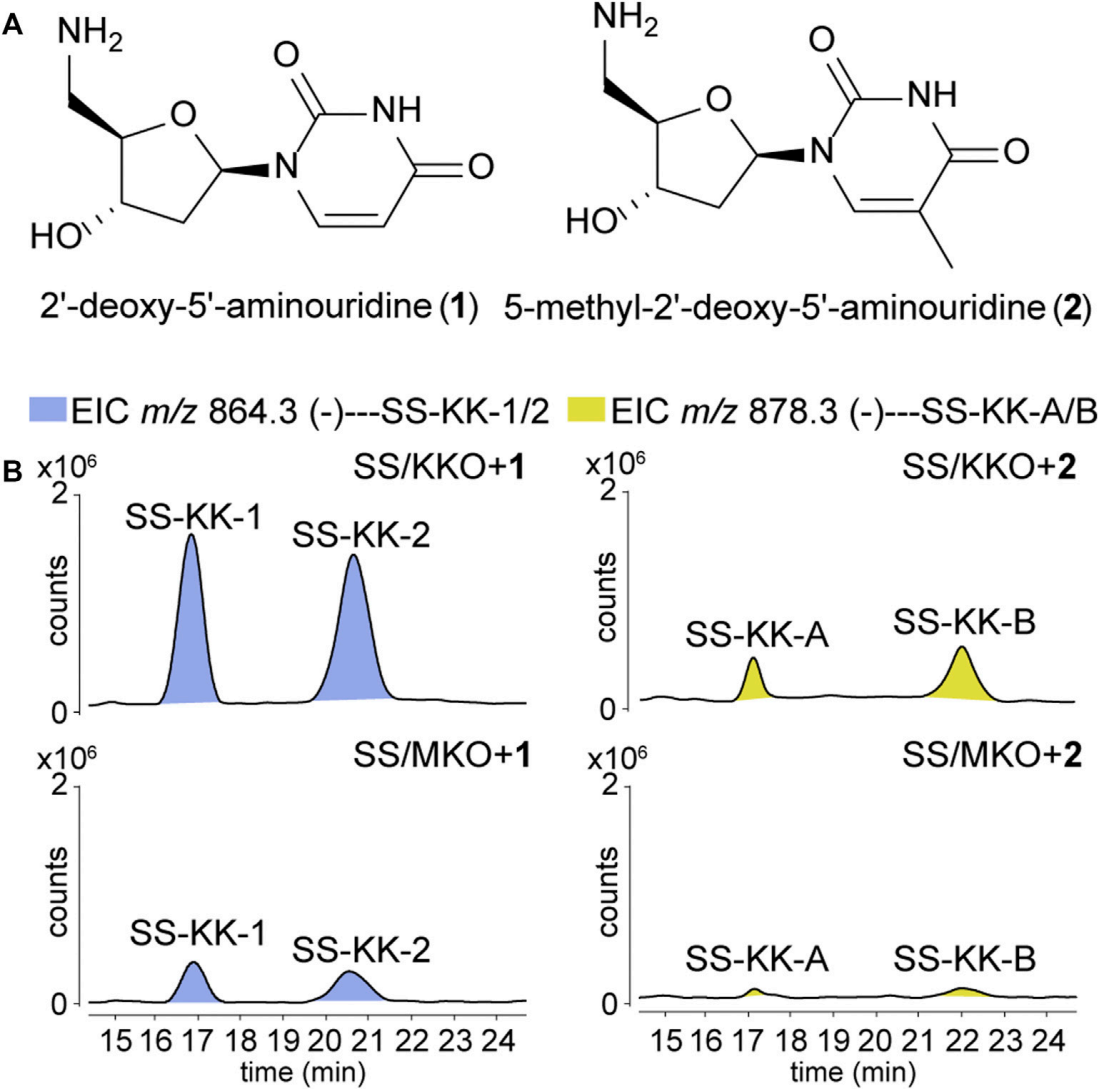
Production of new sansanmycin analogues with 5′-aminouridine modification by mutational biosynthesis. **(A)** Structures of 2′-deoxy-5′-aminouridine (**1**) and 5-methyl-2′-deoxy-5′-aminouridine (**2**). **(B)** LC-ESI-MS analyses of fermentations fed with different 5′-aminouridine analogues **1** and **2**.

**FIGURE 4:**
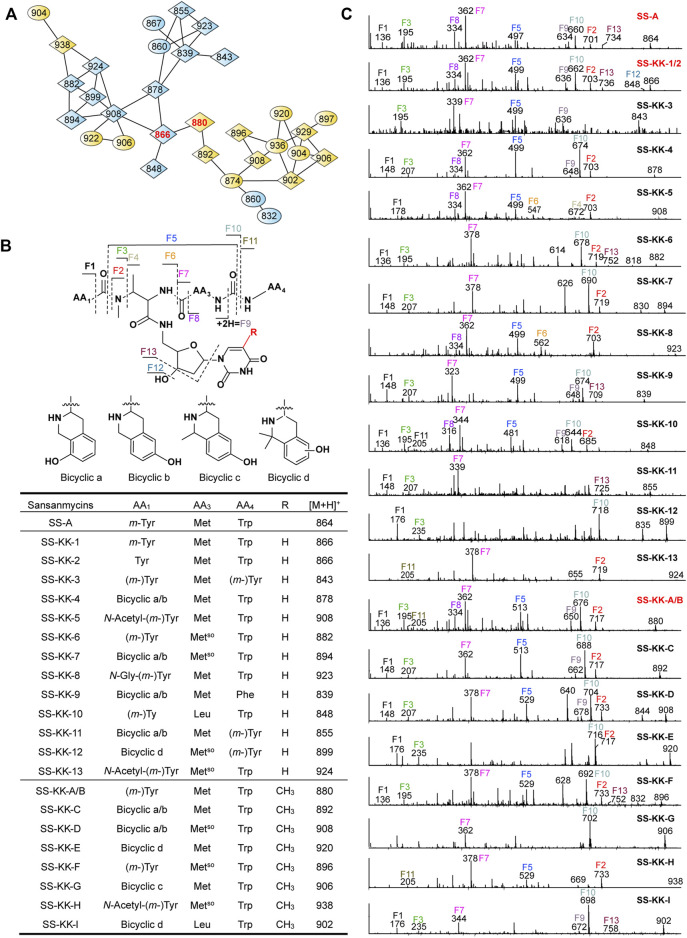
Molecular networking directed discovery of new sansanmycin analogues. **(A)** Molecular network consisting of all parent ions detected by LC–MS/MS in the extract crude of SS/KKO when fed with **1** or **2**. A constellation for potential sansanmycins was picked out using SS-KK-1/2 and SS-KK-A/B (highlight in red) as a probe from the whole molecular network (Supplementary Figure S9) and amplified for displaying. The derivatives produced by feeding SS/KKO with **1** are labeled in blue, while the derivatives produced by feeding SS/KKO with **2** are labeled in yellow. All identified sansanmycin analogues are represented as nodes with diamond symbols. **(B)** The tentative structures of SS-KK-1 to SS-KK-13 and SS-KK-A to SS-KK-I. **(C)** MS/MS analysis for corresponding compounds. The diagnostic fragments are indicated.

To explore the possibility of mutational biosynthesis on modification of 5′-aminouridine moiety, we intended to feed uridine analogues to the knock-out mutants. Besides the natural amide linkage between 5′-aminouridine and tetra/pentapeptides, PacI could also catalyze the formation of an oxoester in the *in vitro* assays for its relaxed substrate specificities (Zhang et al., 2011). Therefore, a series of commercially accessible uridine analogues (Supplementary Figure S8) were firstly fed to SS/KKO. However, no new derivatives were detected in the LC-MS/MS analysis, potentially due to the low efficiency of SsaI (the homologue of PacI) in catalyzing the formation of ester bonds *in vivo*. Then 5′-aminouridine analogues were taken into our consideration and only two commercial compounds were available, 2′-deoxy-5′-aminouridine (1) and 5-methyl-2′-deoxy-5′-aminouridine (**2**) both with the *R*-configuration of the chiral C-4' (Figure 3A). The two 5′-aminouridine analogues 1 and 2 (final concentration of 3 mM) were respectively fed to both SS/MKO and SS/KKO mutants. The fermentation broths were analyzed by extracting ion chromatograms of target compounds derived from SS-A biosynthetic pathway. It was found that two peaks (Figure 3B), with identical MS/MS fragmentations (Figure 4C), appeared in each of the two cultures fed with **1** and **2**. The new derivatives generated from feeding substrate **1** were named SS-KK-1 and SS-KK-2, while those produced from feeding substrate **2** were named SS-KK-A and SS-KK-B.

The molecular weight of SS-KK-1 and SS-KK-2 showed 2 Da larger than that of SS-A, while the molecular weights of **1** is 2 Da larger than that of original 5′-aminouridine moiety, suggesting that 5′-aminouridine of SS-KK-1 and SS-KK-2 were replaced by **1**. This hypothesis was supported by MS/MS spectra (Figure 4C), in which the characteristic fragment ions containing 5′-aminouridine portion, including F5, F6, F12, F13, were 2 Da larger than that of SS-A, and other characteristic fragment ions were consistent with those of SS-A. The F1 ion at *m/z* 136, corresponding to immonium ions of *meta*-tyrosine or tyrosine (Tyr), together with the F2 ion at *m/z* 703, corresponding to the loss of an *m*-Tyr or Tyr residue from *N*-terminus, inferred that SS-KK-1 and SS-KK-2 hold an *m*-Tyr or Tyr at AA_1_ position as found in SS-A (Xie et al., 2007) and SS MX-2 (Shi et al., 2016) respectively.

The molecular weight of **2** was 14 Da larger than that of **1**. Similarly, the quasi molecular of SS-KK-A/B were also 14 Da larger than that of SS-KK-1/2, indicating that the fed precursor **2** was incorporated into the structures of SS-KK-A/B. This inference was supported by the corresponding tandem mass spectrum fragments, in which the characteristic fragment ions containing 5′-aminouridine moiety, including F2, F5, F9, F10, were found to be 14 Da larger than that of SS-KK-1/2, while other characteristic fragment ions were consistent with those of SS-KK-1/2 (Figure 4C). Therefore, it is speculated that SS-KK-A/B are similar to SS-KK-1/2 except for the replacement of 5′-aminouridine with **2**.

The production of SS-KK-1/2 and SS-KK-A/B indicates that feeding 5′-aminouridine analogues to knockout strains SS/MKO and SS/KKO can afford new derivatives. Among them, SS/KKO showed a higher yield of new derivatives (Figure 3), indicating it is a better host for expanding the structural diversity of 5′-aminouridine by mutational biosynthesis. The above results suggest the potential of using mutational biosynthesis for production of 5′-aminouridine-modified sansanmycin derivatives.

### 2.3 New sansanmycin analogues discovered by molecular networking

The method of tandem mass-based molecular networking was previously established for rapid identification of sansanmycin analogues (Jiang et al., 2018). Here, we applied this method for analyzing sansanmycin derivatives produced by the above two cultures fed with precursors **1** and **2**, respectively. Through this analysis, secondary metabolites showing similar fragmentation patterns in tandem mass spectra were grouped into multiple sub-networks (Supplementary Figure S9). Using SS-KK-1/2 and SS-KK-A/B as probes, a sub-network consisting of over 30 nodes was discovered in the molecular networks of extracts from the two cultures (Figure 4A; Supplementary Figure S9). The visualization of metabolites from the two cultures in different colors allowed us to easily distinguish the source of the molecules (Figure 4A; Supplementary Figure S9). Further manual analysis of the MS/MS data corresponding to these molecules reveals that the uridine moiety of the molecules from the culture fed with precursor **1** have altered from the original 5′-aminouridine moiety to **1**. Similarly, the molecules from the culture fed with precursor **2** are found to bear the unit of **2** in place of the original 5′-aminouridine moiety in sansanmycin (detailed analyses are shown in Supplementary Results).

By closer inspection of their MS/MS spectra, we found that, in addition to the alterations in the 5′-aminouridine, the amino acids comprising the pseudopeptide chain also exhibit diversity. Due to the susceptibility of these molecules to cleavage at peptide bonds, analysis of their MS/MS data can provide a clear inference of the types and positions of amino acids in the pseudopeptide chain (Figure 4 and Supplementary Results). For instance, the fragment losses for M-F10 indicate the molecular weight of the *C*-terminal amino acid residue (AA_4_). A mass loss of 204 Da for M-F10 (SS-KK-1/2, SS-KK-4, SS-KK-6, SS-KK-7, SS-KK-10, SS-KK-A/B to SS-KK-G and SS-KK-I) suggests the presence of Trp at AA_4_. Similarly, a shortage of 165 Da indicates that Phe is at AA_4_ (SS-KK-9), while 181 Da (SS-KK-12) points to *m*-Tyr/Tyr ((m-)Tyr) in the place of AA_4_. Therefore, by analyzing the MS/MS fragments and comparing them with those of known sansanmycins (Jiang et al., 2018), the structures of additional eighteen new sansanmycin analogues (SS-KK-3 to SS-KK-13 and SS-KK-C to SS-KK-I) were tentatively deduced (Figure 4 and Supplementary Results).

Due to the limited production of many sansanmycin analogues, their structures can only be deduced through MS/MS data. The molecular networking approach, based on MS/MS data, provides valuable clues towards discovering more 5′-aminouridine-modified sansanmycin derivatives.

### 2.4 Isolation and structural determination of new sansanmycin analogues

To further characterize the structures deduced by MS/MS analysis of sansanmycin derivatives and determine their antimicrobial activity, SS/KKO fermentations fed with **1** and **2** were scaled up. The main components were isolated as previously described (Xie et al., 2007; Xie et al., 2014), and then further purified by semi-preparative HPLC to give SS-KK-1 (2.1 mg), SS-KK-2 (0.8 mg), SS-KK-3 (0.9 mg) and SS-KK-C (0.4 mg). The purity of these compound monomers was determined by HPLC (Supplementary Figures S10–13). Subsequently, the 1D and 2D NMR spectra of SS-KK-1 were collected in DMSO-*d*_6_ to aid in structural elucidation. However, due to the limited quantity of SS-KK-2, SS-KK-3 and SS-KK-C, their structures could not be further confirmed by NMR methods.

The NMR spectra in DMSO-*d*_6_ for SS-KK-1 were very similar to those of SS-A (Xie et al., 2007), except the signals from sugar moiety. Further analysis of these different signals disclosed that the fed 2-deoxy-5′-aminouridine in SS-KK-1 (*δ*H 6.05 for H-sugar-1, 2.02 for H-sugar-2, 3.99 for H-sugar-3, 3.66 for H-sugar-4, 3.21 for H-sugar-5) was in place of 5′-aminouridine in SS-A, which was further confirmed by the sequential ^1^H–1H COSY correlations of H-sugar-1/H-sugar-2/H-sugar-3/H-sugar-4/H-sugar-5. Interpretation of the 1D and 2D NMR spectra (Supplementary Figures S14–19 and Supplementary Table S1) confirmed the proposed structure which holds an *m*-Tyr at the *N*-terminus.

Based on above MS/MS analysis, SS-KK-1 and SS-KK-2 produced the same fragmentations and was expected to hold an *m*-Tyr or Tyr at *N*-terminus, respectively. As *m*-Tyr was confirmed in the SS-KK-1, Tyr was inferred at the *N*-terminus of SS-KK-2.

### 2.5 Antibacterial activity and stability of new sansanmycin analogues

The antibacterial activity of sansanmycin analogues SS-KK-1, SS-KK-2, SS-KK-3, and SS-KK-C was tested against a range of bacteria (Table 1), including both Gram-negative and Gram-positive bacteria, as well as standard strain of *M. tuberculosis* H37Rv, and clinically isolated strains of *M. tuberculosis* S17, M6600, M3551 and M9483. Among them, M6600, M3551, and M9483 are multidrug-resistant strains, resistant to INH at 0.1 μg/mL and RIF at 1 μg/mL. Unexpectedly, both SS-KK-1 and SS-KK-C lost their antibacterial activity against *P. aeruginosa*, *M. tuberculosis*, and even *E. coli ΔtolC*. SS-KK-3 showed the same antibacterial activity as SS-A against *E. coli ΔtolC* and *M. tuberculosis* strains including H37Rv and clinically isolated, drug-sensitive and multidrug-resistant strains. Interestingly, although SS-KK-2 lost its inhibitory activity against *P. aeruginosa*, it showed the best inhibition activity against *E. coli ΔtolC* compared with SS-KK-3 and SS-A. The bioactivity of our newly obtained SS-KK-2 and SS-KK-3, which do not contain the 2′-OH group, suggests that it is not essential for their activity.

**TABLE 1:** Activities of sansanmycin analogues.

Compounds	MIC (μg/mL)							
	*E. coli* ΔtolC	*P. aeruginosa* 11	*M. Phlei*	*M. tuberculosis*				
				H37Rv	S17	M6600	M3551	M9483
SS-KK-1	>32	>32	>32	>64	>64	>64	>64	>64
SS-KK-2	1	>32	>32	32	32	64	64	32
SS-KK-3	4	32	64	16	16	16	16	16
SS-KK-C	>32	>32	>32	>64	>64	>64	>64	>64
SS-A	4	16	64	16	16	16	16	16
streptomycin	2	8	16					
INH				0.03	0.03	R	R	R
RIF				0.125	0.25	R	R	R
LZD				0.25	0.25	S	S	S

*M. tuberculosis* H37Rv, standard strain; S17, M6600, M3551, and M9483 are clinical isolates of *M. tuberculosis*. INH, isoniazid; RIF, rifampicin; LZD, linezolid. M6600, M3551, and M9483, multidrug-resistant strains, resistant to INH, at 0.1 μg/mL and RIF, at 1.0 μg/mL, sensitive to LZD, at 2.0 μg/mL.

**FIGURE 5:**
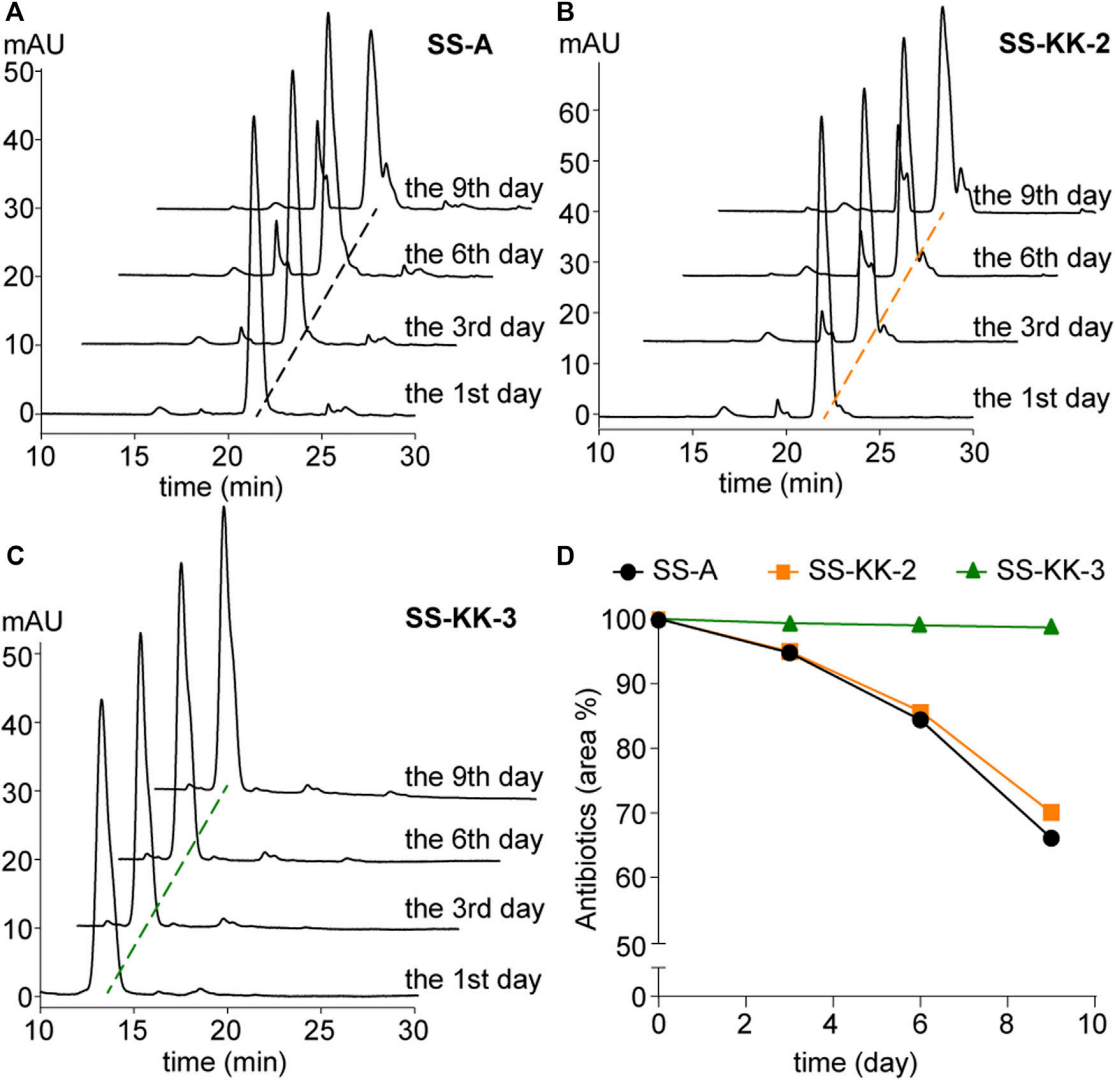
Stability of SS-A, SS-KK-2 and SS-KK-3. **(A–C)** HPLC analysis of SS-A, SS-KK-2 and SS-KK-3 at indicated days in KH_2_PO_4_ buffer (pH 6.0) at room temperature. **(D)** The changes of the level of SS-A, SS-KK-2 and SS-KK-3 over time. All samples were analyzed by HPLC and quantified according to the areas of peaks. Each sample underwent three parallel repeats. SS-A, black line; SS-KK-2, orange line; SS-KK-3, green line.

Poor stability is one of the factors hindering the development of sansanmycin analogues into drug candidates. Thus, we conducted stability experiments followed the method reported previously (Shi et al., 2016) on the two active sansanmycin analogues, SS-KK-2 and SS-KK-3 (Figure 5). These experiments were carried out at room temperature with the parent compound SS-A as a control. When subjected to these conditions, the residual quantities of SS-A were depleted below 70% after 9 days of incubation, whereas SS-KK-2 showed a slightly higher level of stability than SS-A. SS-KK-3 exhibited minimal degradation and substantially greater stability than both SS-A and SS-KK-2. As SS-KK-3 displayed antimicrobial activity comparable to that of SS-A, it is a more promising lead compound for anti-TB drug development.

## 3 Discussion

To expand the structural diversity of 5′-aminouridine moiety of sansanmycin through biosynthetic methods, the function of *ssaM* and *ssaK* as biosynthetic genes for 5′-aminouridine moiety of sansanmycins was firstly validated *in vivo*. The production of hydrated SS-A in the *ssaM*-deletion mutant confirmed the role of *ssaM* as a dehydratase. Unfortunately, due to the low yield of hydrated SS-A, the pure compound could not be obtained for activity assay. Preliminary attempt of feeding easily accessible uridine analogues containing 5′-OH *in vivo*, which can form ester bonds with tetra/pentapeptides catalyzed by PacI *in vitro* (Zhang et al., 2011), did not result in the expected derivatives by mutational biosynthesis. This could be due to either the varying efficiency of SsaI, which is the homologue of PacI, between *in vivo* and *in vitro* conditions, or to the larger substrate tolerance of PacI compared to SsaI. As expected, 5′-aminouridine analogues were shown to be readily incorporated into sansanmycin biosynthetic pathway in SS/KKO and SS/MKO mutant, with higher effectiveness in SS/KKO mutant. To the best of our knowledge, it is the first report to modify 5′-aminouridine of UPAs and obtained a range of 5′-aminouridine-modified derivatives through mutational biosynthesis, which confirms that SS/KKO obtained in this study is a good host for enhancing the structural diversity of 5′-aminouridine of UPAs. In contrast to tedious and challenging chemical synthesis, employing SS/KKO to increase the structural diversity of 5′-aminouridine via mutasynthesis is more efficient and effective.

he 5′-aminouridine moiety of UPAs, as present in other classes of naturally occurring nucleoside inhibitors such as muraymycin, caprazamycin and capuramycin, resembles the uridine moiety of UDP-MurNAc pentapeptide, the natural substrate of MraY. Recent structural biological data of MraY-inhibitor complex also showed a common feature of the uridine binding pocket of naturally occurring nucleoside inhibitors including caprazamycin, capuramycin, and mureidomycin (Mashalidis et al., 2019). The chemically synthesized dihydropacidamycin D resulting from reduction of the chemically unstable 4′-exo double bond of ribosyl moiety showed similar antibacterial activity to that of pacidamycin D (Boojamra et al., 2001). Lemoine et al. further synthesized a series of dihydropacidamycin D derivatives by chemically modifying the uracil ring of 5′-aminouridine, however, none of these derivatives were capable of inhibiting MraY, highlighting the crucial role of uracil for UPAs’ activity (Lemoine et al., 2002). These early discoveries were also consistent with recent structural biological data of MraY-inhibitor complex, which revealed that the uridine pocket of MraY has a certain level of spatial tolerance. Moreover, compared to the ribosyl moiety, the modifications in the uracil moiety are more likely to interfere with the interactions between the residues forming the uridine pocket of MraY, leading to the loss of inhibitory effect (Mashalidis et al., 2019). In this study, SS-KK-C, an analogue obtained from the culture fed precursor **2** with a methyl modification on uracil, also lost its antibacterial activity against *P. aeruginosa*, *M. tuberculosis*, and even *E. coli ΔtolC*.

Previous chemically synthesized 5′-aminouridine-modified derivatives have always contained a 2′-OH on the nucleoside ribosyl group (Figure 1) (Inukai et al., 1993; Boojamra et al., 2001; Okamoto et al., 2012; Tran et al., 2017; Mashalidis et al., 2019; Niro et al., 2020; Tran et al., 2021). By generating a series of 5′-aminouridine analogues that lacks 2′-OH for the first time, this study demonstrated that the presence of 2′-OH is dispensable for the bioactivity of UPAs. In previous reports, the *R*-stereochemistry at the C-4′ position of dihydropacidamycin has been considered as playing a significant role in maintaining antibacterial activity (Boojamra et al., 2001; Tran et al., 2017). However, we noticed that SS-KK-1 with the *R*-stereochemistry at the C-4′ position of the ribose attached to the same tetrapeptide backbone as sansanmycin A completely losses antibacterial activity. Further inspection of the structure of uridine unit disclosed that although the new dihydro UPA derivatives obtained in this study share the *R*-stereochemistry at the C-4′ position of the ribose with the chemically synthesized dihydro UPAs reported previously (Boojamra et al., 2001; Tran et al., 2017), their relative configurations differ. The chemically synthesized dihydro UPAs bear an *α*-l-ribose configuration, while the dihydro UPA derivatives in this study show a *β*-d-ribose configuration. It appears that the role of stereochemistry of C-4′ position required further evaluation using more UPA derivatives with structurally diverse uridine.

As uridine moiety is an important part of pharmacophore, further research should be conducted on the structure-activity relationship of the nucleoside part of UPAs. Here we show that mutational biosynthesis offers an efficient and accessible method to expand the structural diversity of 5′-aminouridine moiety of sansanmycin. In the future, by synthesizing structurally varied 5′-aminouridine analogues for mutasynthesis in SS/KKO, the diversity of UPA structures may be further increased, which would provide more valuable insights into the structure-activity relationship of the nucleoside part in UPAs. Additionally, utilizing the biosynthetic method to obtain UPAs with different 5′-aminouridine modifications may be combined with genetically manipulated biosynthesis on the pseudopeptide parts *in vivo*, which will significantly enhance the diversity of UPAs and hold promise for obtaining UPAs with improved activity and/or druggability.

## 4 Conclusion

This study confirmed the functions of SsaM and SsaK in the biosynthesis of 5′-aminouridine of sansanmycin through gene deletion and complementation experiments. Based on this, mutational biosynthesis enriched the diversity of nucleoside moiety of sansanmycins by applying two 5′-aminouridine analogues, especially in *ssaK* knockout strain. Combining manual analysis and molecular networking of MS/MS data, twenty-two new 5′-aminouridine-modified sansanmycin derivatives were identified, among which four were purified and their antibacterial activities were determined. Of note, SS-KK-3 exhibited anti-mycobacterial activity comparable to that of sansanmycin A, even to clinically isolated multidrug-resistant strains of *M. tuberculosis*. Furthermore, SS-KK-3 exhibited substantial improvements in stability compared to sansanmycin A. The enhanced druggability of this compound may spur further research into the development of new anti-TB drug candidates acting against clinically unexplored target MraY.

## 5 Materials and methods

### 5.1 Strains, plasmids and growth conditions

To propagate and transform the sansanmycin-producing strain *Streptomyces* sp. SS, obtained from China Pharmaceutical Culture Collection (CPCC 200442), the strain was grown on solid S5 medium (Wang et al., 2009) at 28°C for sporulation and in liquid fermentation medium (Xie et al., 2007) for sansanmycin production. Mannitol soya flour (MS) agar medium (Kieser et al., 2000) was used for conjugation, and liquid phage medium (Korn et al., 1978) was used for genomic DNA isolation. *Escherichia coli* DH5α was used for general cloning experiments (Sambrook, 2001), while *E. coli* ET12567/pUZ8002 (Kieser et al., 2000) was used for conjugal transfer following established protocols. *E. coli* strains were incubated in Luria–Bertani medium (LB) (Sambrook, 2001) at 37°C. Strains were incubated with apramycin (Am, 50 μg/mL), kanamycin (Km, 25 μg/mL), and chloramphenicol (Cm, 25 μg/mL) when required. The antimicrobial activity of the compounds was tested against *E. coli ΔtolC* mutant, *P. aeruginosa* 11, *M. phlei*, and *M. tuberculosis*. All strains and plasmids used in this study see Table 2.

**TABLE 2:** Strains and plasmids used in this study.

Strains/plasmids	Relevant characteristics	References
Strains		
*Streptomyces* sp. SS	Wild-type strain (sansanmycin-producing strain, CPCC200442 from China Pharmaceutical Culture Collection)	Xie et al. (2007)
SS/MKO	*Streptomyces* sp. SS with the in-frame deletion of *ssaM*	This study
SS/KKO	*Streptomyces* sp. SS with the in-frame deletion of *ssaK*	This study
SS/MKO/pL-ssaM	SS/MKO with the expression vector pL-ssaM	This study
SS/KKO/pL-ssaK	SS/KKO with the expression vector pL-ssaK	This study
*Escherichia coli* DH5α	General cloning host	Sambrook (2001)
*Escherichia coli* ET12567/pUZ8002	Strain used for *E. coli*/*Streptomyces* conjugation	Paget et al. (1999)
*Escherichia coli ΔtolC*	Strain for testing antimicrobial activity	Tang et al. (2013)
*Pseudomonas aeruginosa* 11	Strain for testing antimicrobial activity	Xie et al. (2008)
*Mycobacterium phlei*	Strain for testing antimicrobial activity	
*Mycobacterium tuberculosis*	Strain for testing antimicrobial activity	
H37Rv	Standard strain, susceptible to isoniazid and rifampicin	
S17	Clinically isolated strain, susceptible to isoniazid and rifampicin	
M6600	Clinically isolated drug-resistant strain, resistant to isoniazid and rifampicin	
M3551	Clinically isolated drug-resistant strain, resistant to isoniazid and rifampicin	
M9483	Clinically isolated drug-resistant strain, resistant to isoniazid and rifampicin	
Plasmids		
pKCcas9dO	Vector used for the construction of deletion plasmids	He et al. (2015)
pCB003	Vector used as the template for amplifying sgRNA	Jiang et al. (2015)
pKC-M	pKCcas9dO derivative plasmid with the deletion of *ssaM*	This study
pKC-K	pKCcas9dO derivative plasmid with the deletion of *ssaK*	This study
pKC-E	pKCcas9dO derivative plasmid with the deletion of *ssaE*	This study
pSET152	*Streptomyces* integrative vector	Bierman et al., 1992
pL646	pSET152 derivative containing the constitutive promoter *ermE*p*	Hong et al. (2007)
pL-ssaM	pL646 derivative plasmid containing complete coding region of *ssaM*	This study
pL-ssaK	pL646 derivative plasmid containing complete coding region of *ssaK*	This study

### 5.2 Construction of *Streptomyces* sp. SS ssaM and ssaK mutant

The mutant strains *ΔssaM* or *ΔssaK* were generated by CRISPR/Cas9-mediated deletion. The mutant strain *ΔssaE* was also attempted to be constructed using the same method, but the correct strain was not obtained in the end. The pKCcas9dO (He et al., 2015) which comprises a codon-optimized *cas9* for *Streptomyces* was used for gene deletion. To design the sgRNA targeting sequences, the gene sequences of interest were firstly submitted on the website http://www.rgenome.net/cas-designer/, and the output result is a series of sgRNA targeting sequences containing 20 bp located inside the gene of interest. Then, the off-target rate of sgRNA targeting sequences is assessed using CasOT (Xiao et al., 2014) to find sgRNA sequences that can be used to knock out the gene of interest. The sgRNA module plasmid pCB003 was used for amplifying M-sgRNA, K-sgRNA, and E-sgRNA. M-sgRNA, K-sgRNA, and E-sgRNA fragments were amplified with specific forward primers M-sg/K-sg/E-sg (Supplementary Table S2) which contain sgRNA targeting sequences of *ssaM*/*ssaK*/*ssaE*, and general reverse primers P2 (Supplementary Table S2), respectively. Homologous arm flanking *ssaM*, *ssaK* and *ssaE* were amplified from *Streptomyces* sp. SS genomic DNA by PCR with specific primer pairs MP3/MP4 and MP5/MP6, KP3/KP4 and KP5/KP6, and EP3/EP4 and EP5/EP6 respectively. For the deletion plasmid of *ssaM*, the fragment containing M-sgRNA and homologous arms were assembled by overlapping PCR with primer pair M-sg/MP6, then the fragment was cloned into *Spe*I-*Hin*dIII sites of pKCcas9dO to obtain the pKC-M. For the deletion plasmids of *ssaK* and *ssaE*, the fragment containing K-sgRNA or E-sgRNA and corresponding homologous arms were assembled into *Nde*I-*Bam*HI sites of pL646 (Hong et al., 2007) with NEBuilder HiFi DNA Assembly Cloning Kit (New England Biolabs, United States) to obtain the pL-KO and pL-EO, then the fragment obtained by pL-KO and pL-EO digested with *Spe*I/*Hin*dIII was cloned into *Spe*I-*Hin*dIII sites of pKCcas9dO to obtain the pKC-K and pKC-E. The constructed plasmids were introduced into *Streptomyces* sp. SS by conjugation from *E. coli* ET12567/pUZ8002. The resulting mutant strains were designated SS/MKO and SS/KKO, but SS/EKO was not obtained.

### 5.3 Construction of complementary strains

Complementation plasmids for mutant strains *ΔssaM* or *ΔssaK* were constructed using pL646 (Hong et al., 2007) as the vector. The *ssaM* and *ssaK* genes were amplified from the genomic DNA of *S*. sp. SS using ssaM-F/ssaM-R and ssaK-F/ssaK-R primers (Supplementary Table S2), respectively. These genes contained *Nde*I and *Bam*HI restriction sites and were cloned into the T-vector prior to sequencing. After correctly sequencing the *ssaM* and *ssaK* genes, they were ligated into the *Nde*I and *Bam*HI restriction sites of the pL646 (Hong et al., 2007) vector resulting in the plasmids pL-ssaM and pL-ssaK. The recombined plasmids were validated by conjugation transfer into their corresponding knockout strains resulting in complemented strains SS/MKO/pL-ssaM and SS/KKO/pL-ssaK.

### 5.4 Analysis of production of sansanmycins

The methods for fermentation, isolation, and high-pressure liquid chromatography (HPLC) analysis of sansanmycins were conducted in accordance with previously published procedures (Xie et al., 2007; Xie et al., 2014). Cultivation of *Streptomyces* sp. SS and its derivatives proceeded in a 100 mL volume of liquid fermentation medium at 28°C under agitation at 200 rpm. After 2 days of culture, a 5% seed culture was transferred into new fermentation medium and maintained at 28°C for an additional 5 days under agitation at 200 rpm. During the feeding experiment, exogenous substrate (uridine analogue-1 to -12 (Supplementary Figure S8) and 5′-aminouridine analogues **1**/**2** (Figure 3A)) was added to the fermentation medium at a final concentration of 3 mM. The fermented broth was clarified by centrifugation at 4 °C for 20 min at 4,000 rpm, and the supernatant was harvested and subjected to HPLC-MS analysis after extraction with a Sep-Pack C18 cartridge. HPLC-MS analysis was performed on an Agilent 6,410 (Agilent Technologies, United States) equipped with XBridge^®^ C18 column (4.6 × 150 mm, 3.5 μm, Waters, Dublin, Ireland), using a gradient mobile phase that consisted of solvent A (water +0.1% w/v ammonium acetate) and solvent B (100% MeOH). The gradient started from 80:20 solvent A: solvent B and was changed to 40:60 solvent A: solvent B within 30 min. The flow rate was set at 0.8 mL/min.

### 5.5 Tandem mass-based molecular networking

For analyzing sansanmycins through LC-MS/MS, the fermentation broth was enriched using macroporous absorbent resin D4006 (Nankai University Fine Chemical Experiment Factory, Tianjin, China) column. After elution with acetone aqueous solutions of various concentrations, sansanmycins were obtained in 20% and 30% solutions respectively. The crude extract consisting of sansanmycins was acquired after removing the acetone through rotary evaporation. LC-ESI(+)MS/MS data was collected on a Waters ACQUITY UPLC H-Class system (Milford, MA, United States) with a Waters Xevo G2-XS QTof detector (Manchester, United Kingdom). ACQUITY UPLC CSHTM C18 column (1.7 μm, 2.1 × 100 mm, Waters) was employed during UPLC analysis carried out at a flow rate of 0.3 mL/min. Solvent A consisted of 0.1% (w/v) formic acid in water, while Solvent B contained 0.1% (w/v) formic acid in acetonitrile. Over 10 min, mobile phase B increased gradually from 10% to 40%. Mass spectral data were acquired in continuum mode using the fast DDA function. The obtained raw data was converted into.mzML format before undergoing further analysis via the molecular networking tool on the GNPS (global natural product social molecular networking) website (https://gnps.ucsd.edu/ProteoSAFe/static/gnps-splash.jsp). Various parameters defaulted and precursor ion mass was set to 1.00 Da with an MS/MS fragment ion tolerance of 0.02 Da. Finally, results were downloaded and visualized by employing Cytoscape 3.9.1.

### 5.6 Purification and characterization of sansanmycin analogues

Separation and purification of sansanmycin analogues followed the method reported previously (Xie et al., 2014) with some modifications.

Fermentation supernatants were subjected to macroporous absorbent resin D4006 column chromatography. Sansanmycins were eluted in 20% and 30% acetone aqueous solutions and then separately passed through DEAE-Sephadex A-25 chromatograph (GE Healthcare, United States), utilizing Tris-HCl (20 mM, pH 8.5) plus varying concentrations of NaCl, while being monitored by HPLC-UV. The effluent consisting of target compounds was collected and subjected to further purification using preparative HPLC, conducted on a SHIMADZU LC-20 HPLC machine equipped with a diode array detector (DAD). A XBridge^®^ prep C18 column (5 μm, 250 × 10 mm, Waters) was used during preparative HPLC. NMR data from the purified samples were acquired employing Bruker spectrometers and DMSO-*d*6 as solvent.

**SS-KK-1**: white amorphous powder; UV (MeOH) *λ*max (log ε) 216 (4.77), 265 (4.26) nm [α]^20^_D_ −21.05 (c0.19, MeOH).

### 5.7 Antibacterial assay

To determine the inhibition activity of sansanmycin analogues against *M. tuberculosis* H37Rv and clinically isolated strains, a measurement was conducted by the microplate Alamar blue assay, following the method described by (Zhang et al., 2013). The analogues were first dissolved in dimethyl sulfoxide (DMSO) and then serially diluted to final concentrations (μg/mL) ranging from 64 to 0.125 before being tested with 7H9 broth supplemented. INH, RIF and linezolid (LZD) were used as controls for *M. tuberculosis* H37Rv and clinical strains isolated by previous reported method (Hameed et al., 2022). DMSO served as a negative control in these experiments. Minimum inhibitory concentration (MIC) is defined as the lowest concentration of samples that prevents the conversion of the blue color into pink color. Triplicate wells per drug concentration were used.

The microdilution assay recommended by the Clinical and Laboratory Standards Institute (CLSI, 2009, formerly NCCLS) was employed to determine the MICs of other bacterial strains. Strains were cultured in Mueller-Hinton broth (MHB) (Weissauer-Condon et al., 1987) and the final bacterial suspension (in MHB medium) was adjusted to 10^6^ cells/ml. Dilutions of test compounds were substituted with MHB medium as described above. Then, triplicate 100 μL transfers of serial dilutions were placed into 96-well plates, after which 100 μL of bacterial suspension was added to each well. MIC is defined as the lowest concentration of samples that restrains the growth of the test organism detected visually following incubation for 10 h at 37°C. Streptomycin served as a positive control.

### 5.8 Stability determination of sansanmycin analogues

The stability assessment of sansanmycin A and its analogues was conducted using a previously reported method (Shi et al., 2016). Sansanmycins were dissolved in 0.05 M KH_2_PO_4_ buffer (with pH adjusted to 6.0 using NaOH) and incubated at 28 °C for 9 days with testing conducted every 3 days. Each sample underwent three parallel repeats. All samples were analyzed by HPLC and quantified by peak area analysis.

## Data Availability

The GNPS data of this study are available from the corresponding author upon request.

## References

[B1] Bierman M. Logan R. O’Brien K. Seno E. T. Rao R. N. Schoner B. E. (1992). Plasmid cloning vectors for the conjugal transfer of DNA from *Escherichia coli* to *Streptomyces* spp. Gene 116, 43–49. 10.1016/0378-1119(92)90627-2 1628843

[B2] Boojamra C. G. Lemoine R. C. Lee J. C. Léger R. Stein K. A. Vernier N. G. (2001). Stereochemical elucidation and total synthesis of dihydropacidamycin D, a semisynthetic pacidamycin. J. Am. Chem. Soc. 123, 870–874. 10.1021/ja003292c 11456620

[B3] Chatterjee S. Nadkarni S. R. Vijayakumar E. K. Patel M. V. Ganguli B. N. Fehlhaber H. W. (1994). Napsamycins, new Pseudomonas active antibiotics of the mureidomycin family from *Streptomyces* sp. HIL Y-82,11372. J. Antibiot. 47, 595–598. 10.7164/antibiotics.47.595 8040059

[B4] CLSI (2009). “Methods for Dilution antimicrobial susceptibility tests for bacteria that grow aerobically: approved standard—8th Edition,” in CLSI document M07-A8 (Wayne: Clinical and laboratory standards institute).

[B5] Gruschow S. Rackham E. J. Elkins B. Newill P. L. Hill L. M. Goss R. J. (2009). New pacidamycin antibiotics through precursor-directed biosynthesis. Chembiochem 10, 355–360. 10.1002/cbic.200800575 19090518

[B6] Hameed H. M. A. Fang C. Liu Z. Ju Y. Han X. Gao Y. (2022). Characterization of genetic variants associated with rifampicin resistance level in *Mycobacterium tuberculosis* clinical isolates collected in Guangzhou chest hospital, China. Infect. Drug. Resist. 15, 5655–5666. 10.2147/idr.s375869 36193294 PMC9526423

[B7] He H. Zheng G. Jiang W. Hu H. Lu Y. (2015). One-step high-efficiency CRISPR/Cas9-mediated genome editing in *Streptomyces* . Acta. Biochim. Biophys. Sin. 47, 231–243. 10.1093/abbs/gmv007 25739462

[B8] Hong B. Phornphisutthimas S. Tilley E. Baumberg S. Mcdowall K. J. (2007). Streptomycin production by *Streptomyces griseus* can be modulated by a mechanism not associated with change in the adpA component of the A-factor cascade. Biotechnol. Lett. 29, 57–64. 10.1007/s10529-006-9216-2 17120093

[B9] Inukai M. Isono F. Takahashi S. Enokita R. Sakaida Y. Haneishi T. (1989). Mureidomycins A-D, novel peptidylnucleoside antibiotics with spheroplast forming activity. I. Taxonomy, fermentation, isolation and physico-chemical properties. J. Antibiot. 42, 662–666. 10.7164/antibiotics.42.662 2498273

[B10] Inukai M. Isono F. Takatsuki A. (1993). Selective inhibition of the bacterial translocase reaction in peptidoglycan synthesis by mureidomycins. Antimicrob. Agents. Chemother. 37, 980–983. 10.1128/AAC.37.5.980 8517724 PMC187869

[B11] Ióca L. P. Dai Y. Kunakom S. Diaz-Espinosa J. Krunic A. Crnkovic C. M. (2021). A family of nonribosomal peptides modulate collective behavior in *Pseudovibrio* bacteria isolated from marine sponges. Angew. Chem. Int. Ed. Engl. 60, 15891–15898. 10.1002/anie.202017320 33961724 PMC8269750

[B12] Jiang Y. Chen B. Duan C. Sun B. Yang J. Yang S. (2015). Multigene editing in the *Escherichia coli* genome via the CRISPR-Cas9 System. Appl. Environ. Microbiol. 81, 2506–2514. 10.1128/AEM.04023-14 25636838 PMC4357945

[B13] Jiang Z. B. Ren W. C. Shi Y. Y. Li X. X. Lei X. Fan J. H. (2018). Structure-based manual screening and automatic networking for systematically exploring sansanmycin analogues using high performance liquid chromatography tandem mass spectroscopy. J. Pharm. Biomed. Anal. 158, 94–105. 10.1016/j.jpba.2018.05.024 29885606

[B14] Karwowski J. P. Jackson M. Theriault R. J. Chen R. H. Barlow G. J. Maus M. L. (1989). Pacidamycins, a novel series of antibiotics with anti-*Pseudomonas aeruginosa* activity. I. Taxonomy of the producing organism and fermentation. J. Antibiot. 42, 506–511. 10.7164/antibiotics.42.506 2498263

[B15] Kieser T. Bibb M. J. Buttner M. J. Chater K. F. Hopwood D. A. Charter K. (2000). Practical *Streptomyces* genetics. Norwich: John Innes Foundation.

[B16] Koppermann S. Cui Z. Fischer P. D. Wang X. Ludwig J. Thorson J. S. (2018). Insights into the target interaction of naturally occurring muraymycin nucleoside antibiotics. ChemMedChem 13, 779–784. 10.1002/cmdc.201700793 29438582 PMC6019934

[B17] Korn F. Weingrtner B. Kutzner H. J. (1978). “A study of twenty actinophages: morphology, serological relationship and host range,” in Genetics of the actinomycetales. Editors Freerksen E. Tarnok I. Thumin H. (New York: Gustav Fischer Verlag Press), 251–270.

[B18] Lemoine R. C. Magon A. Hecker S. J. (2002). Synthesis of base-modified dihydropacidamycins. Bioorg. Med. Chem. Lett. 12, 1121–1123. 10.1016/s0960-894x(02)00100-2 11909731

[B19] Mashalidis E. H. Kaeser B. Terasawa Y. Katsuyama A. Kwon D. Y. Lee K. (2019). Chemical logic of MraY inhibition by antibacterial nucleoside natural products. Nat. Commun. 10, 2917. 10.1038/s41467-019-10957-9 31266949 PMC6606608

[B20] Michailidou F. Chung C. W. Brown M. J. B. Bent A. F. Naismith J. H. Leavens W. J. (2017). Pac13 is a Small, Monomeric dehydratase that mediates the formation of the 3'-deoxy nucleoside of pacidamycins. Angew. Chem. Int. Ed. Engl. 56, 12492–12497. 10.1002/anie.201705639 28786545 PMC5656905

[B21] Niro G. Weck S. C. Ducho C. (2020). Merging natural products: muraymycin-sansanmycin hybrid structures as novel scaffolds for potential antibacterial agents. Chemistry 26, 16875–16887. 10.1002/chem.202003387 32897546 PMC7756498

[B22] Okamoto K. Sakagami M. Feng F. Togame H. Takemoto H. Ichikawa S. (2012). Total synthesis and biological evaluation of pacidamycin D and its 3'-hydroxy analogue. J. Org. Chem. 77, 1367–1377. 10.1021/jo202159q 22196045

[B23] Paget M. S. Chamberlin L. Atrih A. Foster S. J. Buttner M. J. (1999). Evidence that the extracytoplasmic function sigma factor ςE is required for normal cell wall structure in *Streptomyces coelicolor* A3(2). J. Bacteriol. 181, 204–211. 10.1128/JB.181.1.204-211.1999 9864331 PMC103550

[B24] Ragab A. E. Gruschow S. Tromans D. R. Goss R. J. (2011). Biogenesis of the unique 4',5'-dehydronucleoside of the uridyl peptide antibiotic pacidamycin. J. Am. Chem. Soc. 133, 15288–15291. 10.1021/ja206163j 21861518

[B25] Sambrook J. R. D. (2001). Molecular cloning: a laboratory manual. Cold Spring Harbor, NY: Cold Spring Harbor Laboratory.

[B26] Shi Y. Jiang Z. Lei X. Zhang N. Cai Q. Li Q. (2016). Improving the *N*-terminal diversity of sansanmycin through mutasynthesis. Microb. Cell. Fact. 15, 77. 10.1186/s12934-016-0471-1 27154005 PMC4858918

[B27] Tang X. Gross M. Xie Y. Kulik A. Gust B. (2013). Identification of mureidomycin analogues and functional analysis of an *N*-acetyltransferase in napsamycin biosynthesis. Chembiochem 14, 2248–2255. 10.1002/cbic.201300287 24115404

[B28] Tran A. T. Watson E. E. Pujari V. Conroy T. Dowman L. J. Giltrap A. M. (2017). Sansanmycin natural product analogues as potent and selective anti-mycobacterials that inhibit lipid I biosynthesis. Nat. Commun. 8, 14414. 10.1038/ncomms14414 28248311 PMC5337940

[B29] Tran W. Kusay A. S. Hawkins P. M. E. Cheung C. Y. Nagalingam G. Pujari V. (2021). Synthetic sansanmycin analogues as potent *Mycobacterium tuberculosis* translocase I inhibitors. J. Med. Chem. 64, 17326–17345. 10.1021/acs.jmedchem.1c01407 34845906

[B30] Walsh C. T. Zhang W. (2011). Chemical logic and enzymatic machinery for biological assembly of peptidyl nucleoside antibiotics. ACS Chem. Biol. 6, 1000–1007. 10.1021/cb200284p 21851099 PMC3199363

[B31] Wang L. Hu Y. Zhang Y. Wang S. Cui Z. Bao Y. (2009). Role of *sgcR3* in positive regulation of enediyne antibiotic C-1027 production of *Streptomyces globisporus* C-1027. Bmc. Microbiol. 9, 14. 10.1186/1471-2180-9-14 19159491 PMC2657911

[B32] Weissauer-Condon C. Engels I. Daschner F. D. (1987). *In vitro* activity of four new quinolones in Mueller-Hinton broth and peritoneal dialysis fluid. Eur. J. Clin. Microbiol. 6, 324–326. 10.1007/BF02017630 3622502

[B33] Winn M. Goss R. J. Kimura K. Bugg T. D. (2010). Antimicrobial nucleoside antibiotics targeting cell wall assembly: recent advances in structure-function studies and nucleoside biosynthesis. Nat. Prod. Rep. 27, 279–304. 10.1039/b816215h 20111805

[B34] World Health Organization (2022). Global tuberculosis report 2022. Available at: https://www.who.int/teams/global-tuberculosis-programme/tb-reports/global-tuberculosis-report-2022 (Accessed October 27, 2022).

[B35] Xiao A. Cheng Z. Kong L. Zhu Z. Lin S. Gao G. (2014). CasOT: a genome-wide Cas9/gRNA off-target searching tool. Bioinformatics 30, 1180–1182. 10.1093/bioinformatics/btt764 24389662

[B36] Xie Y. Cai Q. Ren H. Wang L. Xu H. Hong B. (2014). NRPS substrate promiscuity leads to more potent antitubercular sansanmycin analogues. J. Nat. Prod. 77, 1744–1748. 10.1021/np5001494 24964393

[B37] Xie Y. Chen R. Si S. Sun C. Xu H. (2007). A new nucleosidyl‐peptide antibiotic, sansanmycin. J. Antibiot. 60, 158–161. 10.1038/ja.2007.16 17420567

[B38] Xie Y. Xu H. Si S. Sun C. Chen R. (2008). Sansanmycins B and C, new components of sansanmycins. J. Antibiot. 61, 237–240. 10.1038/ja.2008.34 18503203

[B39] Zhang N. Liu L. Shan G. Cai Q. Lei X. Hong B. (2016). Precursor-directed biosynthesis of new sansanmycin analogs bearing para-substituted-phenylalanines with high yields. J. Antibiot. 69, 765–768. 10.1038/ja.2016.2 26905760

[B40] Zhang W. Ntai I. Bolla M. L. Malcolmson S. J. Kahne D. Kelleher N. L. (2011). Nine enzymes are required for assembly of the pacidamycin group of peptidyl nucleoside antibiotics. J. Am. Chem. Soc. 133, 5240–5243. 10.1021/ja2011109 21417270 PMC3071879

[B41] Zhang W. Ostash B. Walsh C. T. (2010). Identification of the biosynthetic gene cluster for the pacidamycin group of peptidyl nucleoside antibiotics. Proc. Natl. Acad. Sci. U.S.A. 107, 16828–16833. 10.1073/pnas.1011557107 20826445 PMC2947877

[B42] Zhang Y. J. Reddy M. C. Ioerger T. R. Rothchild A. C. Dartois V. Schuster B. M. (2013). Tryptophan biosynthesis protects mycobacteria from CD4 T-cell-mediated killing. Cell 155, 1296–1308. 10.1016/j.cell.2013.10.045 24315099 PMC3902092

[B43] Zumla A. Nahid P. Cole S. T. (2013). Advances in the development of new tuberculosis drugs and treatment regimens. Nat. Rev. Drug. Discov. 12, 388–404. 10.1038/nrd4001 23629506

