# Supplementary material for: Expanding structural diversity of 5′-aminouridine moiety of sansanmycin via mutational biosynthesis
Source: Front Bioeng Biotechnol. 2023 Oct 30;11:1278601. doi: 10.3389/fbioe.2023.1278601 (PMC10643210; doi:10.3389/fbioe.2023.1278601)
Supplement: Supplementary file 1 [file DataSheet1.pdf]

Supplementary Material

Table of Contents

1 Supplementary Results..3

1.1 Structures identification of new sansanmycin analogues by molecular networking and manual analysis of MS/MS spectra..3

2 Supplementary Tables and Figures 5

Table S1. ^{1}H NMR (600 MHz) and ^{13}C NMR (150 MHz) Data for SS-KK-1. 5

Table S2. PCR primers used in this study...6

Figure S1. Biosynthetic scheme for 4',5'-enamide-3'-deoxyuridine..8

Figure S2. Sequence alignment of SsaM and PacM. 9

Figure S3. Sequence alignment of SsaK and PacK..10

Figure S4. Sequence alignment of SsaE and PacE...11

Figure S5. Verification of *ssaM* knockout strains by PCR. 12

Figure S6. Verification of *ssaK* knockout strains by PCR...13

Figure S7. The (+)-ESI-MS/MS data of parent ion peak (*m/z* 882) of hydrated SS-A. 14

Figure S8. The structures of uridine analogues fed to SS/KKO...15

Figure S9. Molecular network consisting of all parent ions detected by LC–MS in the extract crude of SS/KKO when fed with **1** or **2**. 16

Figure S10. HPLC-DAD-MS chromatogram for the purity determination of SS-KK-1. 17

Figure S11. HPLC-DAD-MS chromatogram for the purity determination of SS-KK-2. 18

Figure S12. HPLC-DAD-MS chromatogram for the purity determination of SS-KK-3. 19

Figure S13. HPLC-DAD-MS chromatogram for the purity determination of SS-KK-C........20

Figure S14. ^{1}H NMR spectrum of SS-KK-1 (600 MHz, DMSO-d_6)....................................21

Figure S15.^{13}C NMR spectrum of SS-KK-1 (150 MHz, DMSO-d_6)....................................22

Figure S16. H-H COSY NMR spectrum of SS-KK-1 (DMSO-d_6)..23

Figure S17. HSQC spectrum of SS-KK-1 (DMSO-d_6)...24

Figure S18. HMBC spectrum of SS-KK-1 (DMSO-d_6)...25

Figure S19. Selected 2D NMR correlations for SS-KK-1. 26

References...27

1 Supplementary Results

1.1 Structure identification of new sansanmycin analogues by molecular networking and manual analysis of MS/MS spectra

1.1.1 New sansanmycin derivatives generated by feeding precursor **1**

SS-KK-3 have the same AA_1 and AA_3 with SS-KK-1/2 based on their consistent characteristic fragment ions of F5 and F9, but with a total mass weight 23 Da lower than that of SS-KK-1, implying that the AA_4 of SS-KK-3 is (*m*-)Tyr, which has a molecular weight of 23 Da lower than that of tryptophan (Trp).

SS-KK-4, SS-KK-5 and SS-KK-8 were found that only AA_1 was altered compared to SS-KK-1/2 based on the same characteristic fragment ions of F2, F5, F7, and F8. The AA_1 of SS-KK-4 was 12 Da larger than that of *m*-Tyr, indicating the presence of a bicyclic a/b which was previously found in sansanmycin K and J (Xie et al., 2014). In SS-KK-5, AA_1 was 42 Da larger than *m*-Tyr, possibly due to an additional acetylation, resulting in *N*-Acetyl-(*m*-)Tyr. The AA_1 of SS-KK-8 was 57 Da larger than that of *m*-Tyr, which is speculated to be an additional glycine (Gly) substitution resulting *N*-Gly-(*m*-)Tyr.

SS-KK-6 has a molecular weight of 16 Da larger than SS-KK-1, suggesting that they may have one more oxygen atom. The AA_3 of SS-KK-1 might be replaced by oxidized methionine (Met^{SO}) rather than methionine (Met). This hypothesis was also supported by the characteristic losses of methylsulfonyl group (64 Da) of *m/z* 818 of M–64 and *m/z* 614 of F10–64.

SS-KK-7 exhibited molecular ion $[M+H]^+$ at *m/z* 894, 16 Da more than that of SS-KK-4. All the characteristic fragments related to AA_3 (F2, F5, and F10) are also 16 Da more than those in SS-KK-4, suggesting that it has an additional oxygen atom compared to SS-KK-4 at the AA_3 position.

The quasi-molecular ion $[M+H]^+$ of SS-KK-9 at *m/z* 839 was 39 Da lower than that of SS-KK-4. The tandem mass spectrometry analysis confirmed the presence of a consistent characteristic fragment ion at *m/z* 674 (F10), indicating that the AA_4 was changed from Trp in SS-KK-4 to Phe in SS-KK-9.

The quasi-molecular of SS-KK-10 was 18 Da lower than that of SS-KK-1. After in-depth analysis, it was found that the change occurs in AA_3 due to the F7 and F10 of SS-KK-10 both lower than those of SS-KK-1. Therefore, AA_3 was speculated to be leucine (Leu) with a molecular weight 18 Da lower than that of Met.

SS-KK-11 has a molecular weight of 854, 12 Da larger than that of SS-KK-3. While both compounds showed consistent F7 ion at *m/z* 339, the F3 ion at *m/z* 207 in SS-KK-11 was 12 Da greater than the F3 ion at *m/z* 195 observed in SS-KK-3. Thus, it can be inferred that AA_1 of SS-KK-11 was bicyclic a/b.

The tandem mass spectrum of SS-KK-12 differs significantly from the resolved sansanmycins. It was speculated that AA_3 of SS-KK-12 is Met^{SO} because of the appearance of diagnostic fragments ion of *m/z* 835 (M-64). Characteristic fragment of F1 ion at *m/z* 176, corresponding to the residue of AA_1, was 28 Da larger than that of bicyclic a/b, suggesting that the AA_1 of SS-KK-12 is bicyclic d with two more methyl groups compared to bicyclic a/b. Based on this, AA_4 was presumed to be *m*-Tyr/Tyr ((*m*-)Tyr) according to the quasi-molecular ion of SS-KK-12. The remaining characteristic fragment ions also supports this inference.

The quasi-molecular of SS-KK-13 was 16 Da larger than that of SS-KK-5. Combining with diagnostic fragment ion at *m/z* 655 (F2-64), the AA_3 SS-KK-13 was inferred to be Met^{SO}.

1.1.2 New sansanmycin derivatives generated by feeding precursor **2**

The molecular weight of **2** was 14 Da larger than that of **1**. Similarly, the quasi molecular of SS-KK-C, SS-KK-F and SS-KK-H were 14 Da larger than that of SS-KK-4, SS-KK-6 and SS-KK-12, respectively. It is speculated that SS-KK-C, SS-KK-F and SS-KK-H share the same amino acids as SS-KK-4, SS-KK-6 and SS-KK-12, respectively, only uridine was replaced by **2**. This inference was also supported by the corresponding tandem mass spectrum fragments.

SS-KK-D has a molecular weight of 16 Da larger than SS-KK-C, suggesting the AA_3 of SS-KK-D may be Met^{so}, which is 16 Da larger than Met. This hypothesis was also confirmed by characteristic fragments of *m/z* 844 (M-64) and *m/z* 640 (F10-64).

The molecular weight of SS-KK-E was 28 Da higher than that of SS-KK-C, likely due to two additional methyl substitutions on AA_1, which suggests AA_1 is bicyclic d. The consistent fragments of F2 ions at *m/z* 717 indicates the change only occurs on AA_1, while the characteristic fragment of F1 ion at *m/z* 176, corresponding to the residue of bicyclic d, further supports this inference.

The quasi-molecular of SS-KK-G was 14 Da larger than that of SS-KK-C, possibly because of an extra methyl substitution. Both compounds share the same AA_3 and AA_4 as evidenced by the identical fragments of F7 ion at *m/z* 362. This suggests that the AA_1 in SS-KK-G may be bicyclic c, which has one more methyl substitution than bicyclic a/b, and was previously found in sansanmycins (Jiang et al., 2018).

The quasi-molecular of SS-KK-I showed 18 Da lower than that of SS-KK-E, and it can be inferred that they contain the same AA_1 due to the same ions of *m/z* 176 (F1) and *m/z* 235 (F3). F10 ion of *m/z* 698 in SS-KK-I was 18 Da lower than that of SS-KK-E, indicating that the AA_3 of SS-KK-I should be Leu rather than Met.

2 Supplementary Tables and Figures

Table S1. ^{1}H NMR (600 MHz) and ^{13}C NMR (150 MHz) Data for SS-KK-1.

				Extra signals due to conformers	
**Position***	**Multiplicity**	δ_C	δ_H (*J*, Hz)	δ_C	δ_H (*J*, Hz)
uracil-2	N-CO-N	163.1			
uracil-4	CO-N	150.4			
uracil-5	CH	101.9	5.61, d (8.1)	101.8	5.56, d (8.1)
uracil-6	CH	140.8	7.62, d (8.1)	140.7	7.60, d (8.1)
sugar-1	O-CH-N	84.5	6.06, d (6.6)	84.2	6.05, d (6.8)
sugar-2	CH_2	38.4	2.02, m		
sugar-3	O-CH	70.7	3.99, m	71.1	3.95, m
sugar-4	CH	84.2	3.66, m		
sugar-5	CH_2	40.8	3.21, m		
DABA-1	CO-N	175.1			
DABA-2	CH	55.3	4.47, d (9.08)	55.3	4.45, d (9.09)
DABA-3	CH	51.5	4.08, m		
DABA-4	CH_3	14.5	0.73, d (6.48)	13.6	1.03, d (6.48)
DABA-N-CH_3	N-CH_3	27.3	2.59, s	28.2	2.83, s
m-Tyr-1	CO-N	173.8		173.3	
m-Tyr -2	CH	52.4	4.08, m		
m-Tyr -3	CH_2	40.1	2.59, m<br>2.56, m		
m-Tyr-1'	ArC	127.5			
m-Tyr-2'	ArCH	115.1	6.64, s		
m-Tyr-3'	ArC-O	155.9			
m-Tyr-4'	ArCH	115.1	6.63, d (8.4)		
m-Tyr-5'	ArCH	130.4	7.00, m		
m-Tyr-6'	ArCH	130.2	6.92, d (8.1)		
Trp-1	-COOH	174.4			
Trp-2	CH	54.0	4.29, m		
Trp-3	CH_2	27.8	3.08, m<br>3.02, m		
Trp-2'	CH	123.6	7.09, s		
Trp-3'	ArC	109.8			
Trp-3a'	ArC	127.5			
Trp-4'	ArCH	118.5	7.50, d (7.9)		
Trp-5'	ArCH	118.3	6.94, m		
Trp-6'	ArCH	120.8	7.03, m		
Trp-7'	ArCH	111.2	7.30, d (8.1)		
Trp-7a'	ArC	136.0			
ureido	N-CO-N	no signal			
Met-1	CO-N	171.9			
Met-2	CH	52.6	4.18, m		
Met-3	CH_2	32.7	1.76, m<br>1.67, m		
Met-4	CH_2	29.5	2.35, m		
Met-S-CH_3	CH_3	14.7	1.97, s		

The spectra were recorded in DMSO-d_6. The chemical shifts (δ) are given in ppm.

*Abbreviation for the structure units are: Trp = tryptophan, DABA = 2-amino-3-methyl-aminobutyric acid, Met = methionine

Table S2. PCR primers used in this study.

Name	Sequence (5'-3')	Purpose
M-sg	TCAGTCCTAGGTATAATACTAGTCTATTTCGGCGCGATGGAGAGTTTTAGAGCTAGAAA	Using for amplifying sgRNA fragment targeting *ssaM*
P2	CTCAAAAAAAGCACCGACTCGG	
MP3	TGAAAAAGTGGCACCGAGTCGGTGCTTTTTTTGAGCACCGTCTTCGCCACCCT	Using for amplifying the left homology arm of *ssaM*
MP4	GTGCTTGTGGAATCTGTCGG	
MP5	CACGGTCGAGGAAGCCGACAGATTCCACAAGCACAGGTTCATCAGCGAAAGCG	Using for amplifying the right homology arm of *ssaM*
MP6	CCCAGTCACGACGTTGTAAAACGACGGCCAGTGCCAAGCTTAGGTGCAGGGGCAGGTAG	
ssaM-F	CCCATATGATGACCACGTACCGGCACAC	Using for amplifying the region of *ssaM*
ssaM-R	CGGGATCCTTAGCCGGGGCTCTCGCTT	
K-sg	ATGATTACGAATTCGAGCTCGGTACACTAGTTCCTCGCGCAGTGACCACGCGTTTTAGAGCTAGAAA	Using for amplifying sgRNA fragment targeting *ssaK*
P2	CTCAAAAAAAGCACCGACTCGG	
KP3	TGAAAAAGTGGCACCGAGTCGGTGCTTTTTTTGAGCGGACTTCACCGAGTTCTACA	Using for amplifying the left homology arm of *ssaK*
KP4	AGTCATCAGCAAGTGGTCCC	
KP5	TGCGGAGCGACCTGGGGACCACTTGCTGATGACTGACCCCGAGGGGCTGTTC	Using for amplifying the right homology arm of *ssaK*
KP6	CTTGGGCTGCAGGTCGACTCTAGAGAAGCTTGACTCTTCCGCAGCATCGC	
ssaK-F	CCCATATGATGACTTCTTTCACCGGG	Using for amplifying the region of *ssaK*
ssaK-R	CGGGATCCCTACTGCGCGGGCGCGCC	
E-sg	ATGATTACGAATTCGAGCTCGGTACACTAGTGGATCGCGCTGCAGTACTGGGTTTTAGAGCTAGAAA	Using for amplifying sgRNA fragment targeting *ssaE*
P2	CTCAAAAAAAGCACCGACTCGG	
EP3	TGAAAAAGTGGCACCGAGTCGGTGCTTTTTTTGAGCGCTGTCCTACCGCATCC	Using for amplifying left homology arm of *ssaE*

EP4	CCGCCACAGTGAACTCGTC	
EP5	CACAGTTCCCCTTCGACGAGTTCACTGTGGCGG GTCGAGAACTCCGCCACCCA	Using for amplifying right homology arm of *ssaE*
EP6	CTTGGGCTGCAGGTCGACTCTAGAGAAGCTTCG CTGTCCACGACCACTTC	
MYTKO-F	CGACCACTGGCAGCTCTTCAT	Using for verifying SS/MKO
MYTKO-R	TGCGGTGGGACACCAGGATG	
MYLKO-F	CGCCTCACAGCAAATCGTCTG	Using for verifying SS/MKO
MYLKO-R	CATGCCGTCCTCCATCCAAG	
MYRKO-F	TACAGGGCTGACGACAGGAGG	Using for verifying SS/MKO
MYRKO-R	GGCGATGCTGCGGAAGAGT	
KYTKO-F1	ATCGACCACATCATCAACGACC	Using for verifying SS/KKO
KYTKO-R1	GCTGCCGCTGCTCAGGAA	
KYTKO-F2	GCAGGCACCTCTTCGGCATC	Using for verifying SS/KKO
KYTKO-R2	GCTCGACAGCTCCTGGGACG	

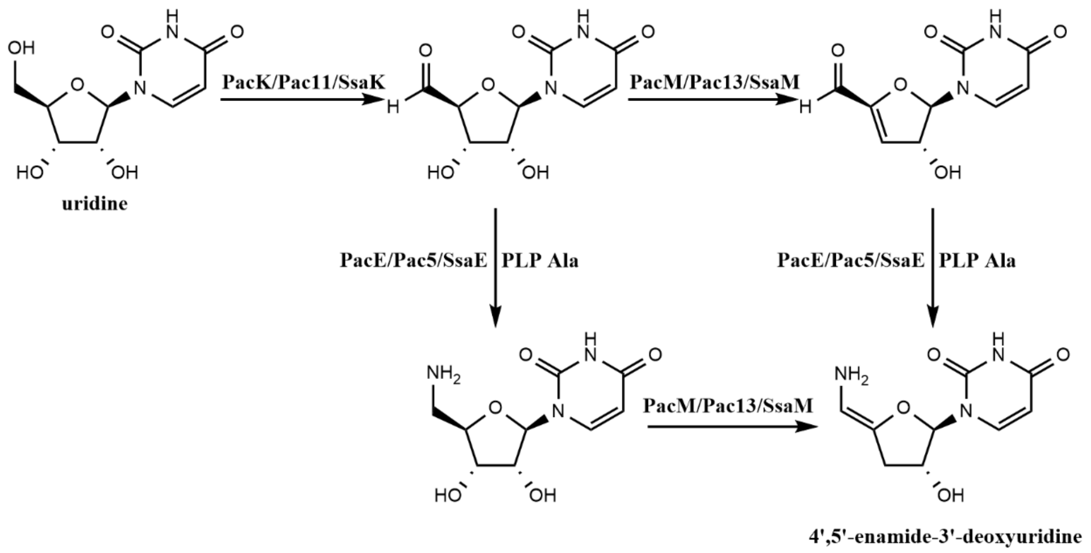

Figure S1. Biosynthetic scheme for 4',5'-enamide-3'-deoxyuridine (Li et al., 2013; Zhang et al., 2010; Zhang et al., 2011; Ragab et al., 2011; Michailidou et al., 2017).

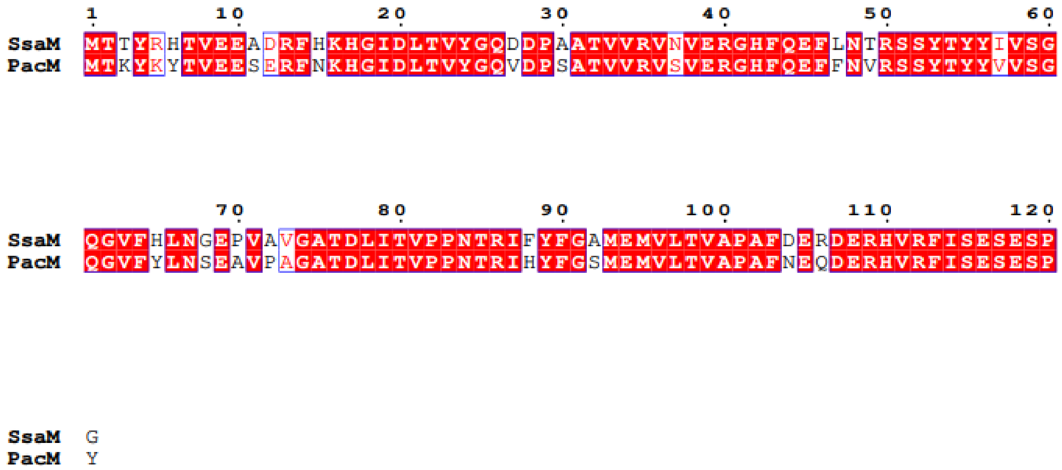

Figure S2. Sequence alignment of SsaM and PacM.

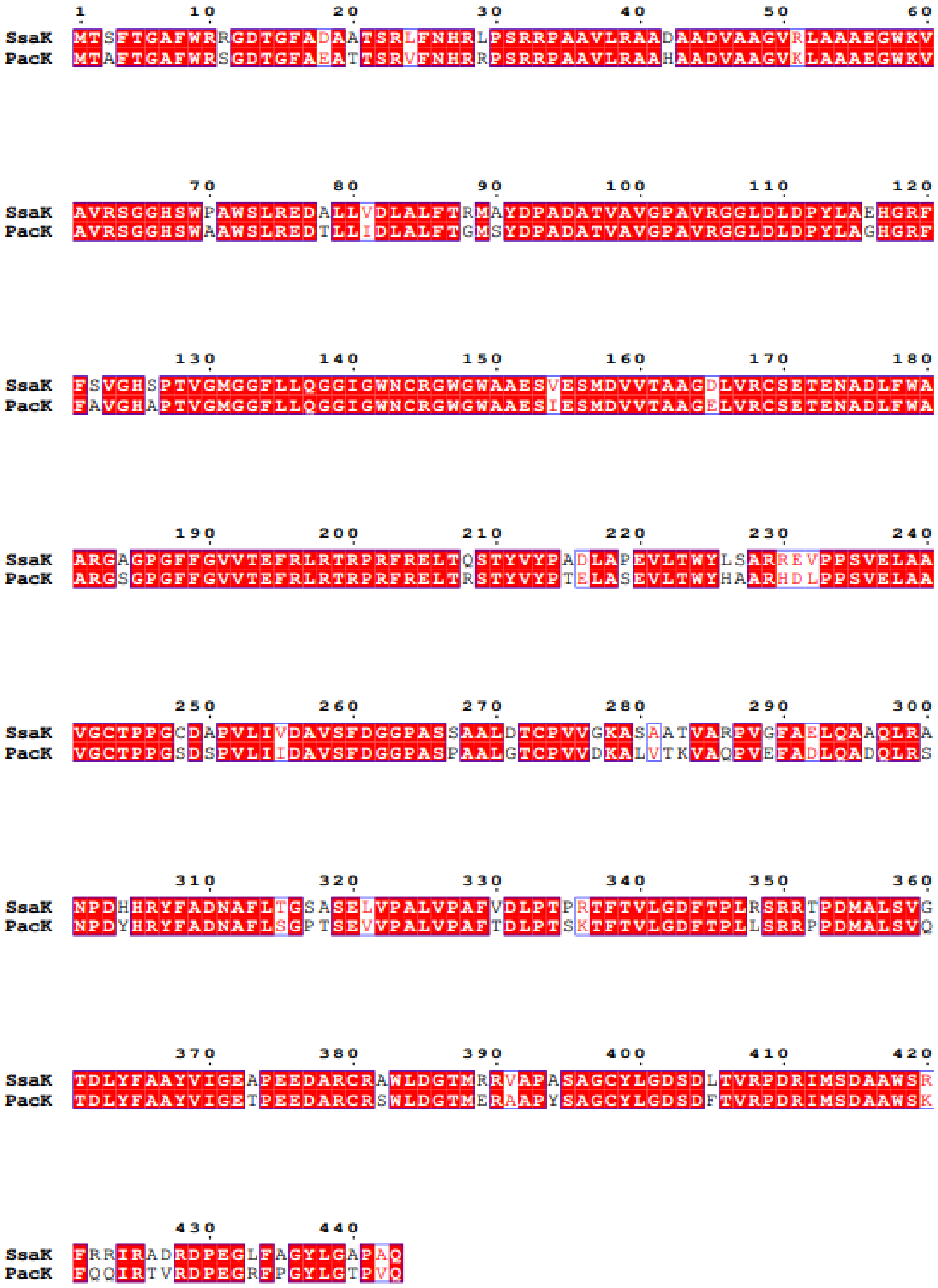

Figure S3. Sequence alignment of SsaK and PacK.

```
        1       10            20        30        40        50
SsaE  MHSSPSTS..SLW......RPW.TPITQQADSMRIVEARGNRVRDADGNWYLDGVSGVLN
PacE  MH..SSTSTSSLWRPWTPITQQAGSLR.......IVEARGNRVRDAEGKWYLDAISGVLN

              60        70        80        90       100       110
SsaE  ASCGHGHPRLIEAASRQFRQLVHYDPMVSSHDPAETLTARLAEILPGELNETVLLNSGSE
PacE  ASCGHGHPRLIEAASRQFEQLVHYDPMVSSHDPAETLASRLAELLPGELNETCLLNSGSE

             120       130       140       150       160       170
SsaE  ATEAAVRIALQYWRNIGEDRNRVITFEAAYHGTTYLAQQLSGLPFTVSEWVPPFPIDHVA
PacE  ATEAALRIALQYWRNIGEDRNRVITFEAAYHGTTYLAQQLSGLPFTASEWAPPFPIEHIA

                  180       190       200       210       220
SsaE  ...LPAAAR..AMRTEESADALVARFARALETGPPAAAVMVEPLLGLGGCVVLPAGFLAR
PacE  LPAAPCAMRTEESADA.....LIALFAKALETGPPAAAVMVEPLLGLGGCVVLPAGF...

       230            240       250       260       270       280
SsaE  LRTLCDQ....HGALLILDEVFCGFGRTGRMFGFDHDGITPDIVTMSKGISGGYLPLAAV
PacE  LAR.LRQLCDQHGALLILDEVFCGFGRTGRMFGFDHDGITPDIVTMSKGISGGYLPLAAV

           290            300       310       320       330
SsaE  TVTSSIKET.....FVREPVVQGLRYGHTTGGHAVAAAVANTVLDIMADEKLVENSATQG
PacE  TVTSSIKETFIREPIAQGLR.....YGHTTGGHAVASNVANAVLDVMADEKLVENSAAQG

      340       350       360       370       380       390
SsaE  AALLDGLRKLESSPLITDVRGLGLVVAVETDSPESGAAITAAAVEAGVMTRHERGVIRIA
PacE  ATLLEGLRKLESSPLITDVRGLGLVVAIEADSPESAADIAAAAAQAGVMTRHERGVIRIA

      400          410       420
SsaE  PPLTLT.AD..DTAEAIEKFGGAVDAVAS.
PacE  PPLTITDDDTAEALQKIS.GAVDIAAAAHR
```

Figure S4. Sequence alignment of SsaE and PacE.

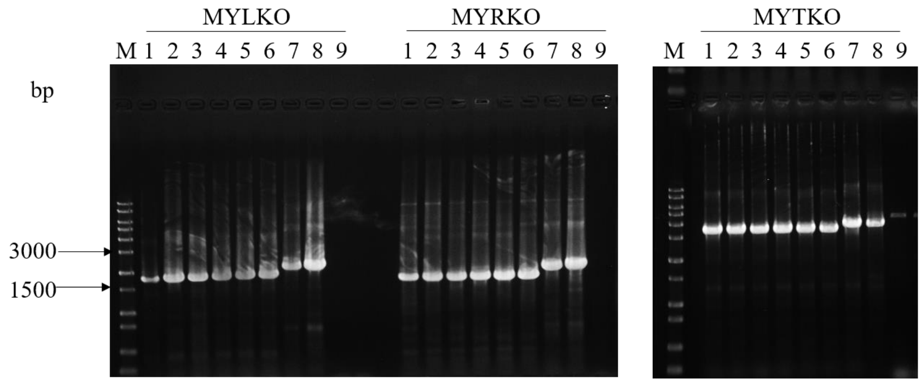

Figure S5. Verification of *ssaM* knockout strains by PCR.

Lane 1 ~ 7: PCR products using SS/MKO genome as templates; Lane 8: PCR products using *S*. sp. SS genome as templates; Lane 9: PCR products using H_2O as templates, negative control. Primers MYLKO-F/MYLKO-R, MYRKO-F/MYRKO-R and MYTKO-F/MYTKO-R were used to amplify 1.7 kb, 1.6 kb and 3.3 kb DNA fragments when using SS/MKO genome as templates and 2.1 kb, 2.0 kb and 3.7 kb DNA fragment when using *S*. sp. SS genome as templates.

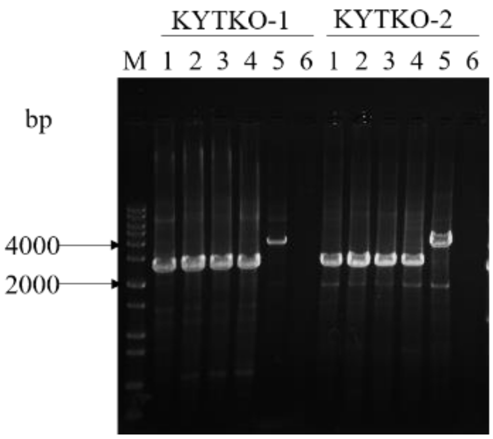

Figure S6. Verification of *ssaK* knockout strains by PCR.

Lane 1 ~ 4: PCR products using SS/KKO genome as templates; Lane 5: PCR products using *S*. sp. SS genome as templates; Lane 6: PCR products using H_2O as templates, negative control. Primers KYTKO-F1/KYTKO-R1 and KYTKO-F2/KYTKO-R2 were used to amplify 2.6 kb and 2.7 kb DNA fragments when using SS/KKO genome as templates and 3.9 kb and 4.0 kb DNA fragment when using *S*. sp. SS genome as templates.

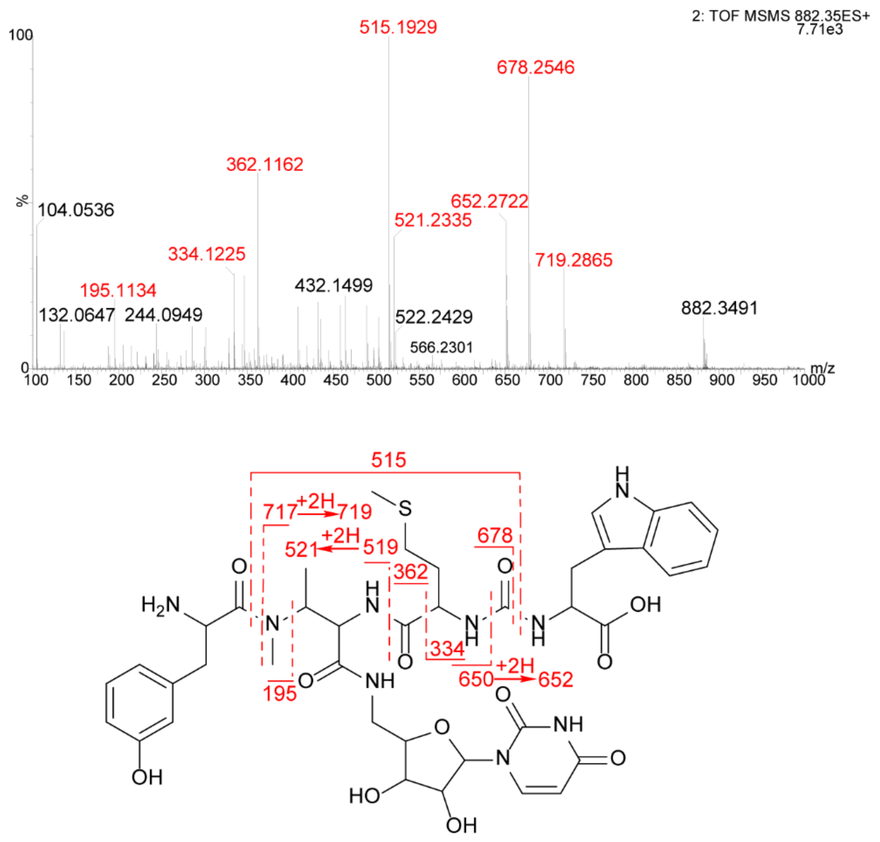

Figure S7. The (+)-ESI-MS/MS data of parent ion peak (*m/z* 882) of hydrated SS-A.

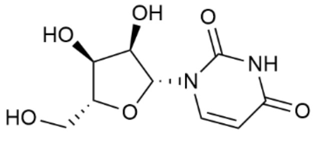

uridine analogue-1

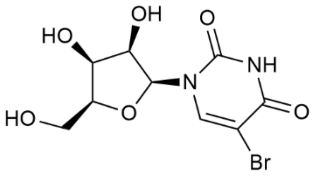

uridine analogue-2

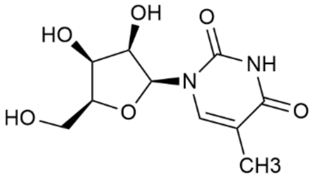

uridine analogue-3

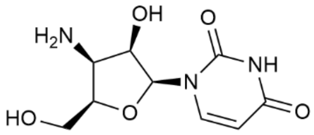

uridine analogue-4

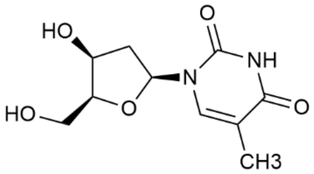

uridine analogue-5

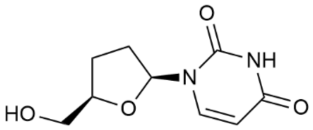

uridine analogue-6

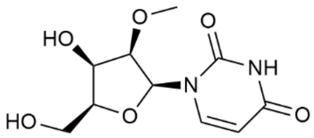

uridine analogue-7

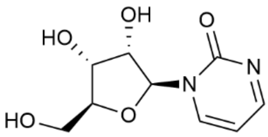

uridine analogue-8

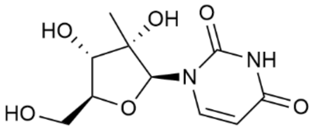

uridine analogue-9

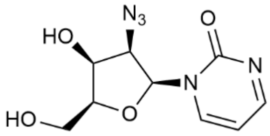

uridine analogue-10

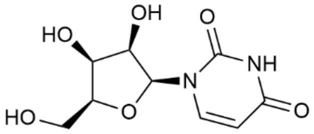

uridine analogue-11

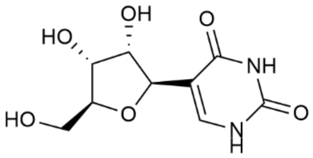

uridine analogue-12

Figure S8. The structures of uridine analogues fed to SS/KKO.

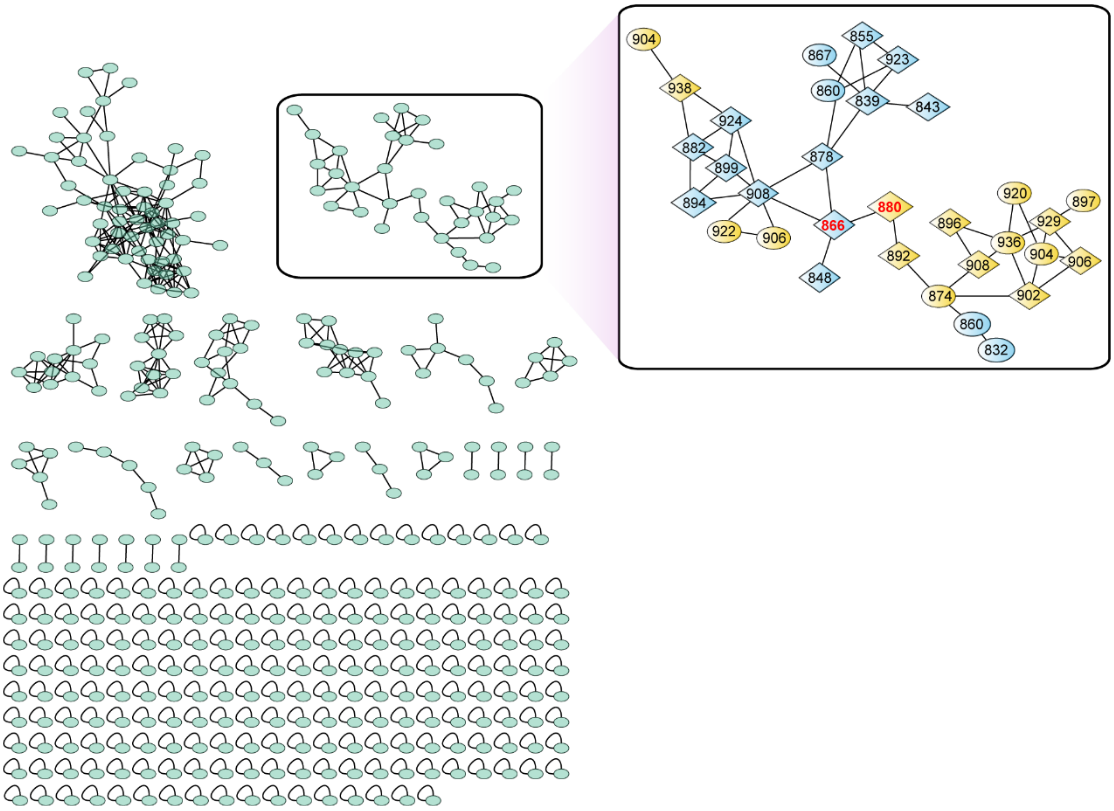

Figure S9. Molecular network consisting of all parent ions detected by LC–MS in the extract crude of SS/KKO when fed with **1** or **2**.

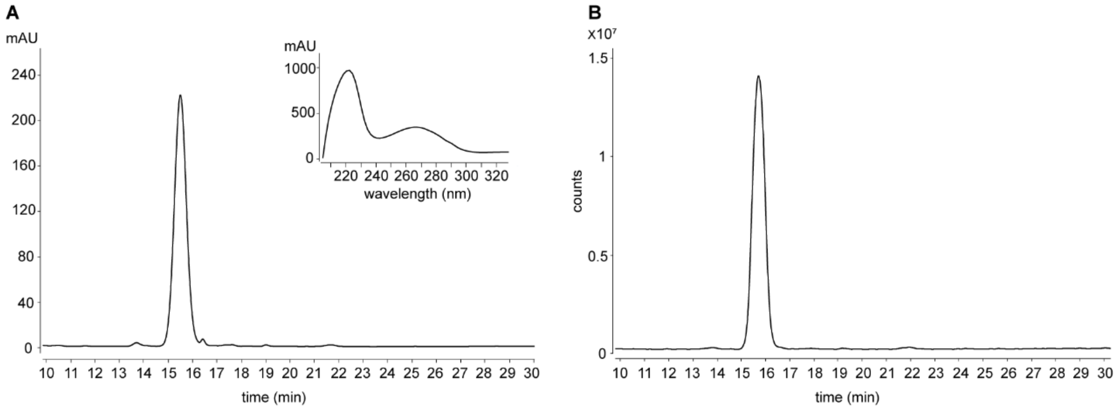

Figure S10. HPLC-DAD-MS chromatogram for the purity determination of SS-KK-1.

(A) Extracted DAD chromatogram at 254 nm and UV absorption spectrum of SS-KK-1. (**B)** Total ion chromatogram (TIC) of SS-KK-1.

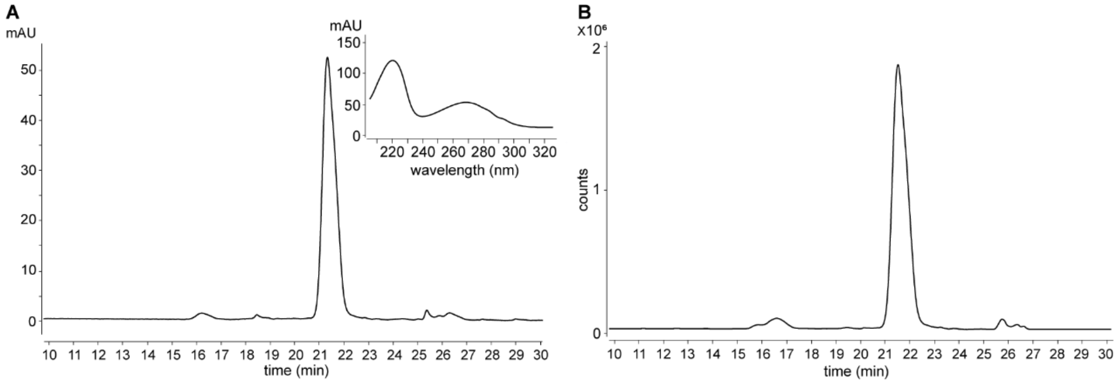

Figure S11. HPLC-DAD-MS chromatogram for the purity determination of SS-KK-2.

(A) Extracted DAD chromatogram at 254 nm and UV absorption spectrum of SS-KK-2. **(B)** Total ion chromatogram (TIC) of SS-KK-2.

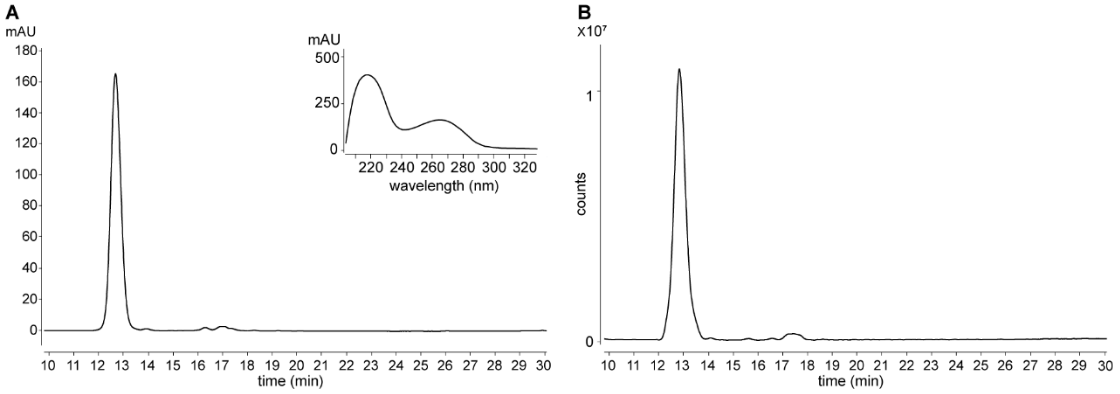

Figure S12. HPLC-DAD-MS chromatogram for the purity determination of SS-KK-3.

(A) Extracted DAD chromatogram at 254 nm and UV absorption spectrum of SS-KK-3. **(B)** Total ion chromatogram (TIC) of SS-KK-3.

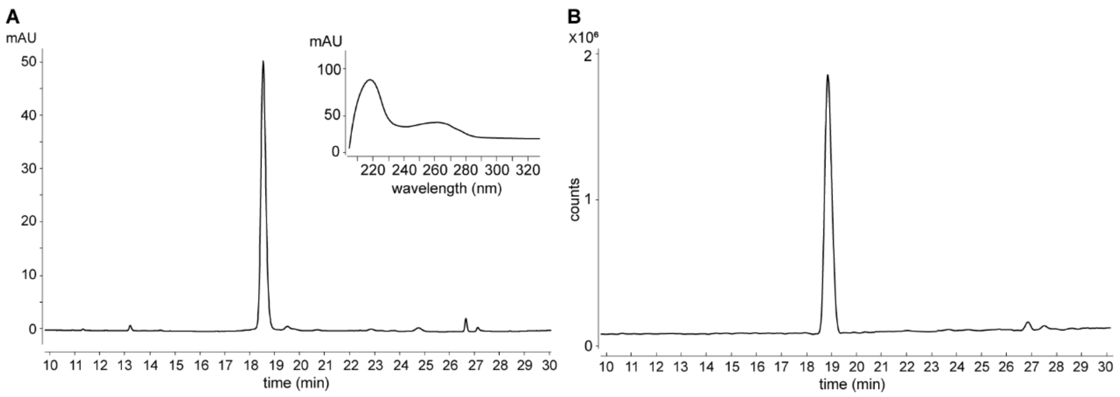

Figure S13. HPLC-DAD-MS chromatogram for the purity determination of SS-KK-C.

(A) Extracted DAD chromatogram at 254 nm and UV absorption spectrum of SS-KK-C. **(B)** Total ion chromatogram (TIC) of SS-KK-C.

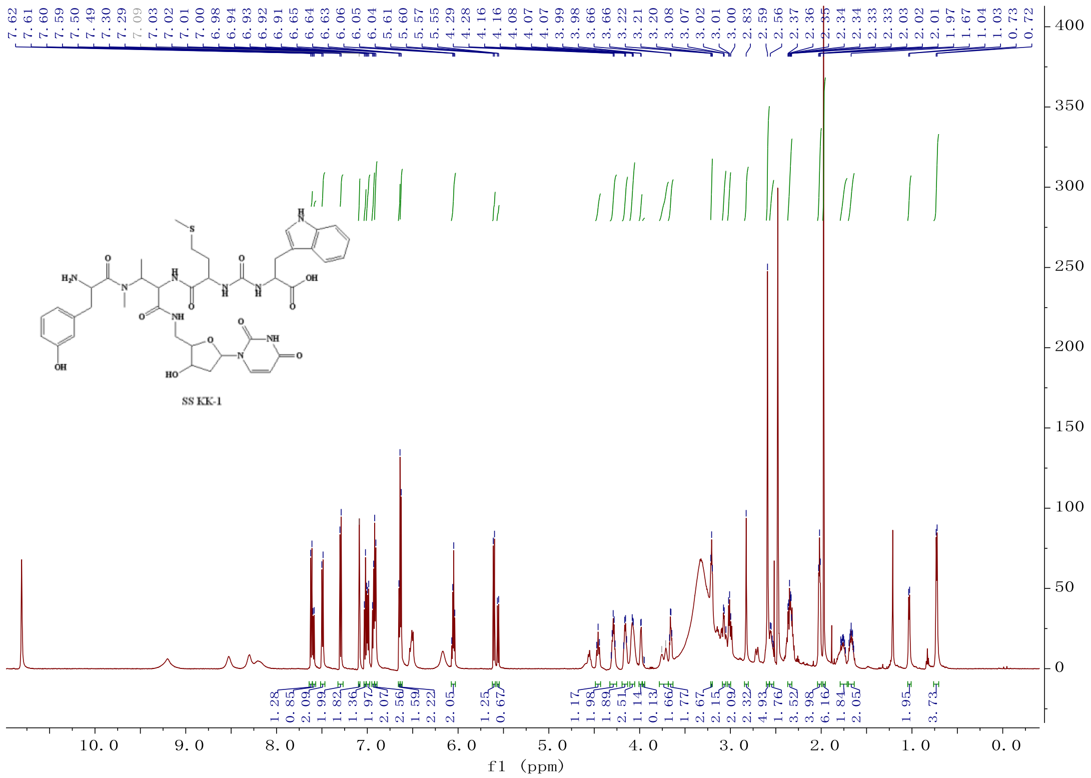

Figure S14. ^{1}H NMR spectrum of SS-KK-1 (600 MHz, DMSO-d_6).

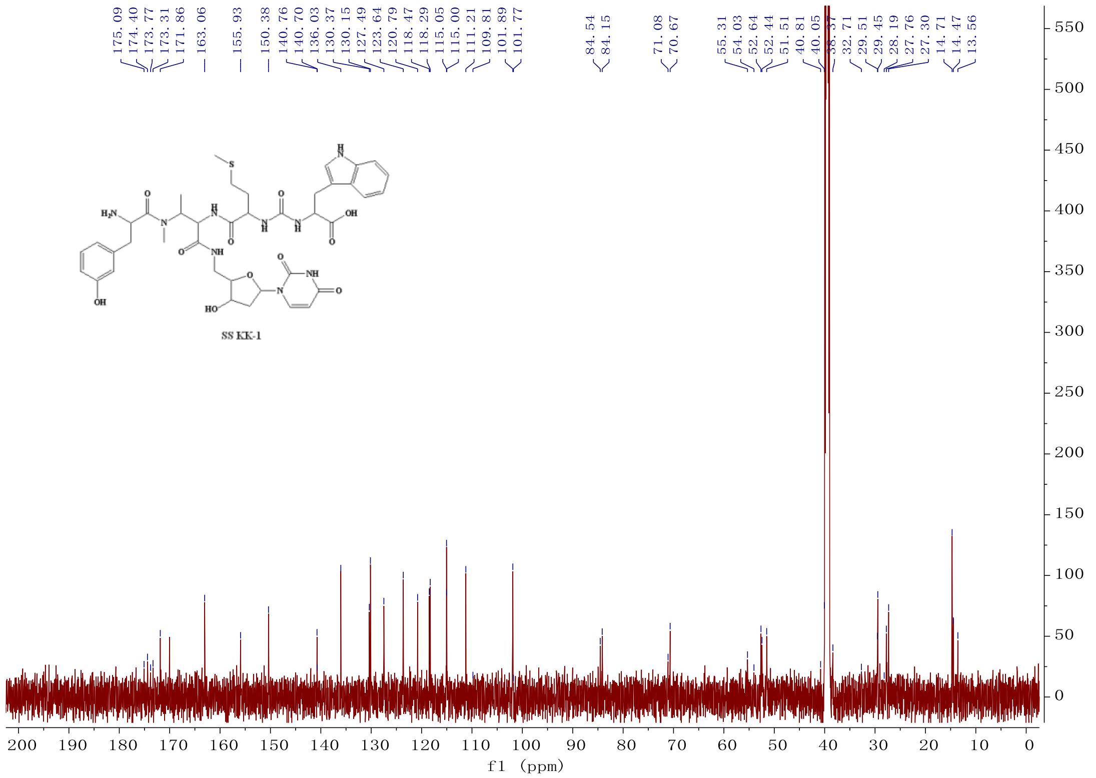

Figure S15.^{13}C NMR spectrum of SS-KK-1 (150 MHz, DMSO-d_6).

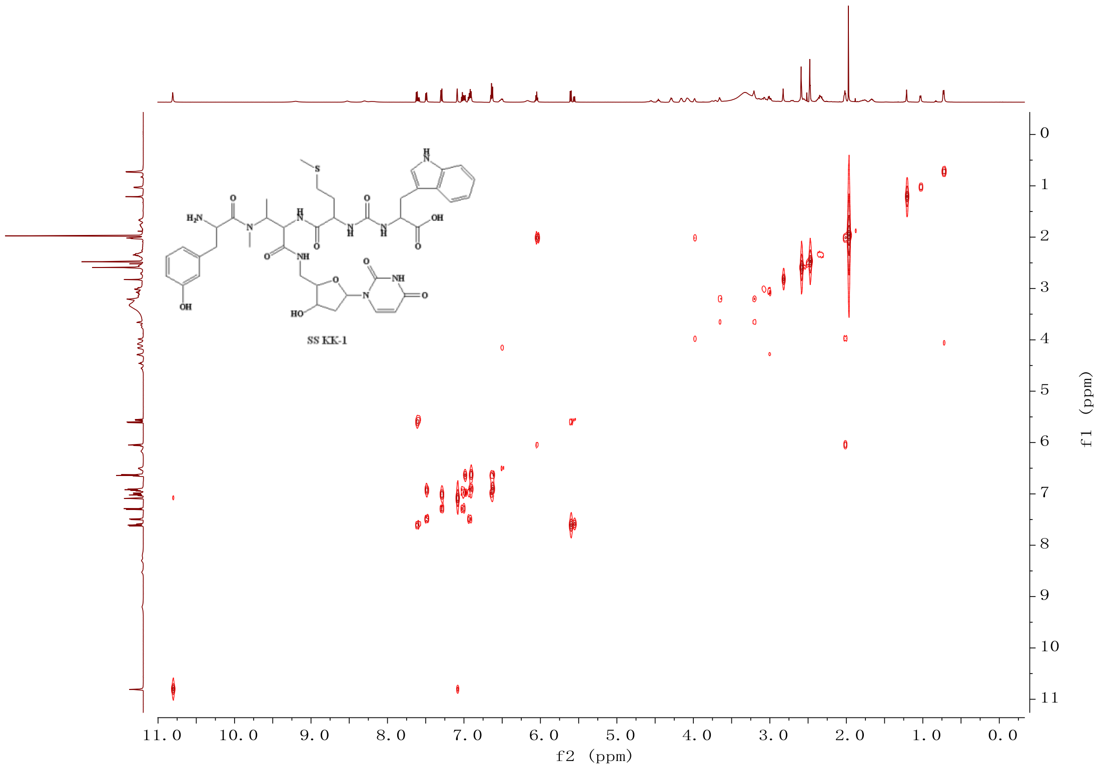

Figure S16. H-H COSY NMR spectrum of SS-KK-1 (DMSO-d_6).

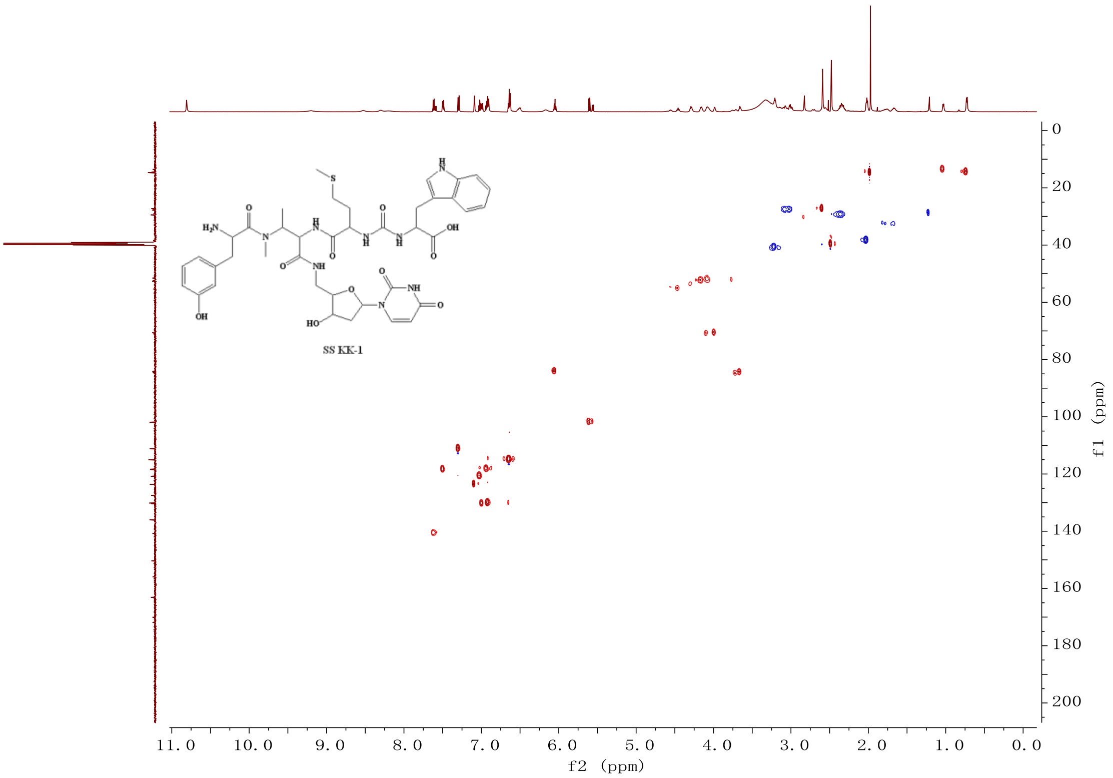

Figure S17. HSQC spectrum of SS-KK-1 (DMSO-d_6).

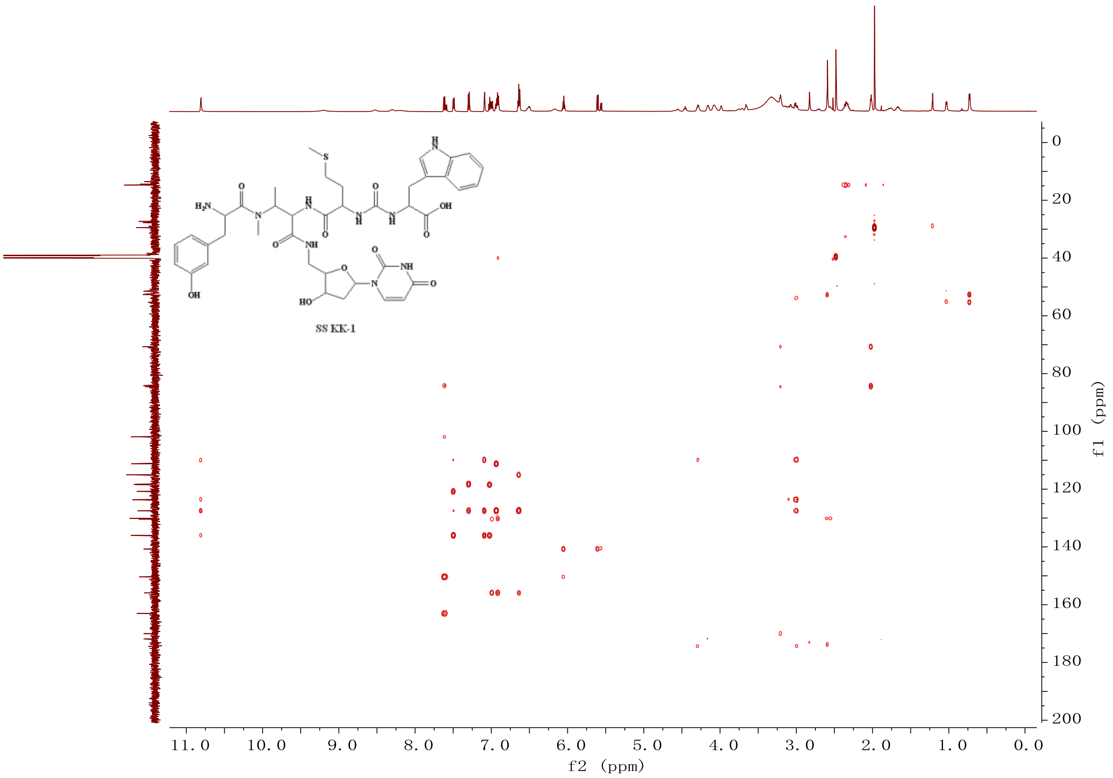

Figure S18. HMBC spectrum of SS-KK-1 (DMSO-d_6).

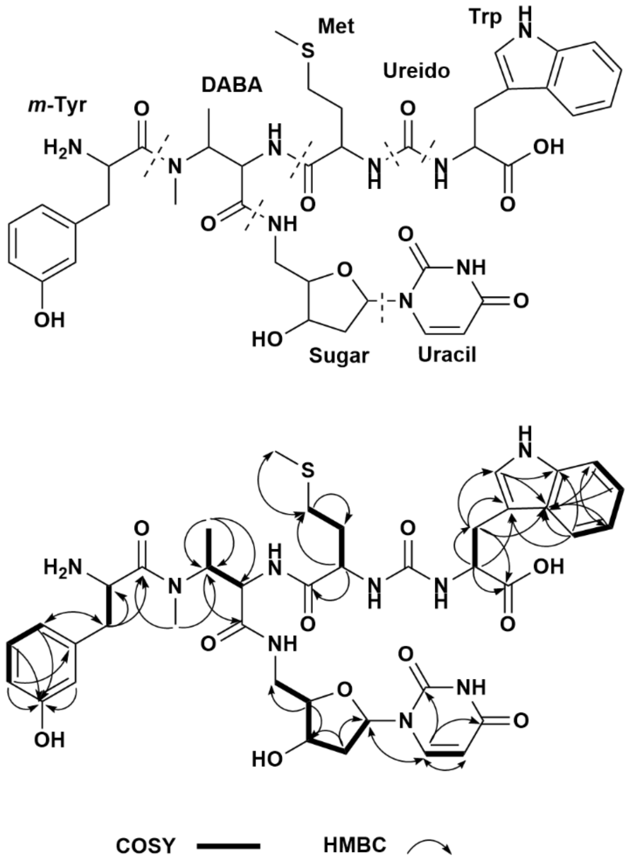

Figure S19. Selected 2D NMR correlations for SS-KK-1.

References

Jiang, Z.B., Ren, W.C., Shi, Y.Y., Li, X.X., Lei, X., Fan, J.H., et al. (2018). Structure-based manual screening and automatic networking for systematically exploring sansanmycin analogues using high performance liquid chromatography tandem mass spectroscopy. *J. Pharm. Biomed. Anal.* 158, 94-105. doi: 10.1016/j.jpba.2018.05.024

Li, Q., Wang, L., Xie, Y., Wang, S., Chen, R., Hong, B. (2013). SsaA, a member of a novel class of transcriptional regulators, controls sansanmycin production in *Streptomyces* sp. strain SS through a feedback mechanism. *J. Bacteriol.* 195, 2232-2243. doi: 10.1128/JB.00054-13

Michailidou, F., Chung, C.W., Brown, M.J.B., Bent, A.F., Naismith, J.H., Leavens, W.J., et al. (2017). Pac13 is a Small, Monomeric dehydratase that mediates the formation of the 3'-deoxy nucleoside of pacidamycins. *Angew Chem. Int. Ed. Engl.* 56, 12492-12497. doi: 10.1002/anie.201705639

Ragab, A.E., Gruschow, S., Tromans, D.R., and Goss, R.J. (2011). Biogenesis of the unique 4',5'-dehydronucleoside of the uridyl peptide antibiotic pacidamycin. *J. Am. Chem. Soc.* 133, 15288-15291. doi: 10.1021/ja206163j

Xie, Y., Cai, Q., Ren, H., Wang, L., Xu, H., Hong, B., et al. (2014). NRPS substrate promiscuity leads to more potent antitubercular sansanmycin analogues. *J. Nat. Prod.* 77, 1744-1748. doi: 10.1021/np5001494

Zhang, W., Ntai, I., Bolla, M.L., Malcolmson, S.J., Kahne, D., Kelleher, N.L., et al. (2011). Nine enzymes are required for assembly of the pacidamycin group of peptidyl nucleoside antibiotics. *J. Am. Chem. Soc.* 133, 5240-5243. doi: 10.1021/ja2011109

Zhang, W., Ostash, B., and Walsh, C.T. (2010). Identification of the biosynthetic gene cluster for the pacidamycin group of peptidyl nucleoside antibiotics. *Proc. Natl. Acad. Sci. USA* 107, 16828-16833. doi: 10.1073/pnas.1011557107
